# Targeted delivery of the PKMYT1 inhibitor RP-6306 mediates PANoptosis in pancreatic cancer via mitotic catastrophe

**DOI:** 10.1038/s41419-025-07835-2

**Published:** 2025-07-15

**Authors:** Jingyun Chen, Jianghao Ren, Chaolei Zhang, Yang Lv, Jingbin Zhou, Weiliang Jiang, Chaojie Huang, Liping Cao

**Affiliations:** 1 Department of General Surgery, Sir Run Run Shaw Hospital, Zhejiang University School of Medicine, Hangzhou, China; 2 Department of Emergency Medcine, Sir Run Run Shaw Hospital, Zhejiang University School of Medicine, Hangzhou, China; 3 Department of Critical Care Medicine, Sir Run Run Shaw Hospital, Zhejiang University School of Medicine, Hangzhou, China; 4 Department of Colon and Rectal Surgery, Sir Run Run Shaw Hospital, Zhejiang University School of Medicine, Hangzhou, China

**Keywords:** Cell delivery, Targeted therapies

## Abstract

Pancreatic ductal adenocarcinoma (PDAC) is a highly malignant tumor often diagnosed in advanced stages due to its subtle early symptoms, making surgical options nonviable and requiring systemic chemotherapy. Current treatments mainly utilize gemcitabine, which provides limited efficacy. PKMYT1, a serine/threonine protein kinase crucial for cell cycle regulation, is overexpressed in PDAC and correlates with poor prognosis. Treatment with the PKMYT1 inhibitor RP-6306 promotes rapid mitotic entry, resulting in DNA damage and mitotic catastrophe, thereby inducing PANoptosis. RP-6306 effectively inhibits PDAC growth in vitro and in vivo, and shows enhanced anti-tumor activity when combined with gemcitabine, also reducing metastasis. However, gemcitabine has notable systemic toxicity. To target cancer cells more specifically, we utilized vesicles derived from cell membranes (BxPC-3M) to deliver a combination of RP-6306 and gemcitabine (GEM + RP-6306@BxPC-3M). This formulation effectively targets homotypic tumor cells and significantly inhibits tumor growth both in vitro and in vivo. These findings highlight the role of RP-6306 in inducing PANoptosis, characterize PANoptosis as a novel form of cell death associated with mitotic catastrophe, and confirm the synergistic antitumor activity of RP-6306 and gemcitabine in PDAC. Moreover, GEM + RP-6306@BxPC-3M exhibits improved safety and enhanced antitumor efficacy.

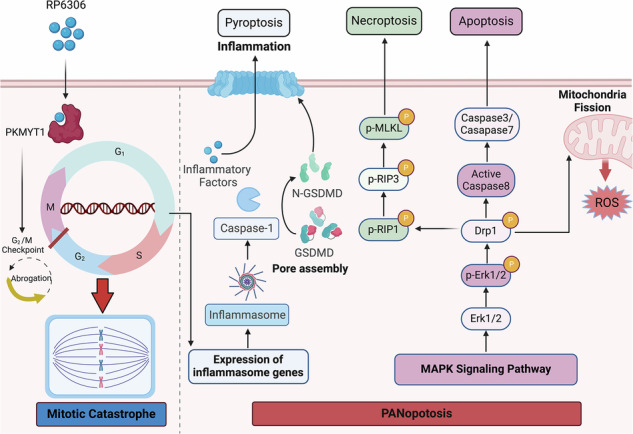

## Introduction

Pancreatic ductal adenocarcinoma (PDAC) is the most prevalent type of pancreatic cancer and is highly malignant. In recent years, immunotherapy and targeted therapy have made some progress in the treatment of pancreatic cancer [1, 2]. Dysregulation of protein kinases plays a crucial role in the pathogenesis of many autoimmune and oncogenic diseases, making protein kinase antagonists vital therapeutic targets [3]. However, the majority of FDA-approved kinase inhibitors target members of the EGFR, VEGFR, and JAK families, with fewer options available for serine/threonine protein kinases [3, 4]. Since the serine/threonine protein kinase family is closely linked to the pathogenesis of pancreatic cancer, inhibitors targeting these kinases hold promise as effective treatments for this disease [4].

The G2 checkpoint in the cell cycle is critical for cancer cells exposed to DNA-damaging agents, providing time for DNA repair before mitosis [5]. Targeting this checkpoint is a viable therapeutic strategy to inhibit cancer growth. WEE1 and PKMYT1 (MYT1) are two protein kinases that regulate the activity of CDK complexes through inhibitory phosphorylation [6]. PKMYT1 is overexpressed in many tumors characterized by replication stress markers, and PKMYT1 inhibitors have shown efficacy in preclinical, in vitro, and in vivo models [6, 7]. RP-6306, a PKMYT1 inhibitor, effectively targets CCNE1-amplified tumor cells and shows enhanced efficacy when combined with agents like hydroxyurea or gemcitabine [8]. A recent Phase I study showed that the PKMYT1 inhibitor lunresertib, combined with FOLFIRI, was effective in a heavily pretreated population of patients with advanced gastrointestinal cancers harboring CCNE1 amplification and FBXW7 mutations.

Cell death, a pivotal strategy in cancer therapy, integrates apoptosis, necroptosis, and pyroptosis—each traditionally viewed as distinct modalities of programmed cell death (PCD). Notwithstanding their diverse signaling pathways, burgeoning evidence underscores a complex interplay among these mechanisms, culminating in the formulation of the PANoptosis paradigm [9]. The growing recognition of PANoptosis for its intricate associations with the tumor immune microenvironment (TIME) and the emergence of oncological resistance positions it as a significant focal point in cancer research [10]. Consequently, the induction of highly immunogenic PANoptosis within tumor matrices emerges as a compelling therapeutic avenue.

Vesicles derived from cell membranes are gaining recognition as innovative vectors for molecular delivery systems. By retaining the intrinsic functionalities and signaling networks of their progenitor cells, these vesicles adeptly traverse a multitude of biological barriers in vivo [11]. Tumor cell-derived vesicles exhibit unique properties in cancer therapy, including persistence, homotypic targeting, and antigen stimulation [12]. The deployment of cell vesicles for the delivery of chemotherapeutic agents, including docetaxel, doxorubicin, and oxaliplatin, has been thoroughly investigated and documented [13–15].

In this study, we identified PKMYT1 as a pivotal therapeutic target for PDAC. PKMYT1 inhibitor (RP-6306), modulates the cell cycle progression and induces PANoptosis in pancreatic cancer cells, thereby inhibiting tumor growth and proliferation both in vitro and in vivo. Furthermore, the combination of RP-6306 and gemcitabine significantly suppressed the growth and metastasis of pancreatic cancer under both conditions. Due to systemic toxicity associated with these drugs, we employed vesicles derived from cell membranes to deliver RP-6306 and gemcitabine. This approach demonstrated enhanced homotypic tumor targeting and more effective suppression of tumor growth in vivo, while also proving to be relatively safe.

## Materials and methods

### Cell lines and cell culture

The human normal pancreatic ductal cell line, hTERT-HPNE, was obtained from Cobioer (Nanjing, China, CBP60875). The pancreatic cancer cell line, PANC-1, was acquired from BnBio (Henan, China, BNCC277096), while BxPC-3, MiaPaCa-2, AsPC-1, and Capan-1 were sourced from the Chinese Academy of Sciences (Shanghai, China). The cell lines hTERT-HPNE, PANC-1, MiaPaCa-2, and Capan-1 were cultured in DMEM (MeilunBio, Liaoning, China, MA0212) with 10% fetal bovine serum (FBS) (Cellmax, Beijing, China, SA211.02) and 1% penicillin/streptomycin (Biosharp, Shanghai China, ML505A). The BxPC-3 and AsPC-1 lines were maintained in RPMI 1640 medium (MeilunBio, China, MA0215). All cell lines were incubated at 37 °C in a 5% CO_2_ humidified atmosphere. Routine tests for microbial contamination, including mycoplasma, were conducted.

### Cell viability assay

The pancreatic cancer cell lines PANC-1, MiaPaCa-2, BxPC-3, and AsPC-1 were seeded in 96-well plates and cultured overnight. Subsequently, the cells were treated with RP-6306 (MedChemExpress, New Jersey, USA, HY-145817A) and gemcitabine (MedChemExpress, USA, HY-17026) in the 96-well plates. Following treatment, CCK-8 reagent (Yeasen, Shanghai, China, 40203ES60) was added, and the plates were incubated for 1 h at 37 °C. Optical density values (OD) at 450 nm was measured using a microplate reader (Thermo Fisher Scientific, Waltham, MA, USA).

### Colony formation assay

Cells were plated at a density of 1 × 10^3^ cells per well in 6-well plates and cultured overnight. The following day, the cells were treated with RP-6306 and/or gemcitabine. After 72 h of treatment, the medium was replaced with either DMEM or RPMI 1640 supplemented with 10% FBS, and the cultures were maintained for approximately 14 days. Cells were then fixed with 4% paraformaldehyde and stained with 0.1% crystal violet solution (Solarbio, Beijing, China, G1064). Colonies were counted using ImageJ software.

### Cell apoptosis assay

Cell apoptosis was assessed using the Annexin V-PE/7-AAD Apoptosis Detection Kit (MultiSciences, Hangzhou, China, AP104) via flow cytometry. Briefly, after treating cells with various concentrations of RP-6306 and/or gemcitabine for 48 h, cells were collected by centrifugation. Annexin V-PE and 7-AAD were then added according to the manufacturer’s instructions and the cells were incubated in the dark at room temperature. Finally, the cells were analyzed using a BD LSR II flow cytometer (BD Biosciences, New Jersey, USA) and FlowJo_V10 software.

### Cell cycle analysis

The Cell Cycle Staining Kit (MultiSciences, China, 70-CCS012) was utilized as per the manufacturer’s recommendations. Cells treated with different concentrations of drugs or for various time periods were collected, and propidium iodide (PI) solution containing RNase A was added. The cells were then incubated in the dark at room temperature. The proportion of cells in G0/G1, S, and G2/M phases was measured using a BD LSR II flow cytometer. Analysis was conducted using FlowJo_V10 software.

### RNA extraction and real-time quantitative PCR (RT-qPCR)

Total RNA was isolated and purified using the Ultrapure RNA Kit (Cwbio, Jiangsu, China, CW0581) according to the manufacturer’s instructions, and the RNA yield was quantified using a NanoDrop spectrophotometer (Thermo, USA). The RNA was then reverse-transcribed into cDNA using the Hifair® II 1st Strand cDNA Synthesis Kit (Yeason, China, 11121ES60), which includes gDNA removal. Subsequent real-time quantitative PCR (RT-qPCR) was performed using Hieff UNICON® qPCR SYBR Green Master Mix (Yeason, China, 11200ES03) on an ABI Q6 real-time PCR System (Applied Biosystems, Thermo Fisher Scientific, USA).

### Western blot

Total cellular proteins were extracted using RIPA buffer (Fdbio, Hangzhou China, FD009) supplemented with a mixture of protease and phosphatase inhibitors (Beyotime, Shanghai, China, P1050). Protein concentrations were quantified using a BCA assay (Beyotime, China, P0010S). Proteins from each sample were separated on SDS-PAGE gels and subsequently transferred onto PVDF membranes. After blocking with BSA, the membranes were incubated overnight at 4 °C with specific primary antibodies, followed by incubation with secondary antibodies at room temperature. Chemiluminescent signals were visualized using a ChemiDoc™ Touch Imaging System (Bio-Rad, California, USA).

### Immunofluorescence staining

Following designated treatments, cells were fixed with 4% paraformaldehyde. Permeabilization was performed with 0.3% Triton X-100 in 3% BSA, after which cells were incubated with primary antibodies for 1 h at room temperature. Following washes with PBS, cells were incubated with appropriate fluorophore-conjugated secondary antibodies for 1 h. Nuclei were stained using DAPI (Abcam, Cambridge, UK, ab104139). All fluorescence images were acquired using a laser scanning confocal microscope (Nikon A1 Ti, Japan) with a 60x oil immersion objective lens.

### Immunohistochemical staining

Immunohistochemical staining was performed using the standard streptavidin-biotin-peroxidase complex method. Briefly, paraffin-embedded tissue sections were dewaxed in a series of alcohols and rehydrated, followed by antigen retrieval by heating the sections in antigen retrieval solution at 55 °C overnight. Endogenous peroxidase activity was blocked, and the slides were incubated with primary antibodies overnight at 4 °C. Detection was accomplished using secondary antibodies (Zsbio, Beijing, China, PV-9001, PV-9002). Sections were counterstained with Gill’s hematoxylin, dehydrated in an ascending series of methanol, cleared in xylene, and mounted under coverslips. Slide images were captured using an automated digital pathology system. Research protocols involving human-derived samples were reviewed and approved by the Research Ethics Committee of Sir Run Run Shaw Hospital affiliated with the School of Medicine, Zhejiang University (20230629-100).

### ROS production assay

For the detection of cytoplasmic reactive oxygen species (ROS), treated pancreatic cancer cells were incubated with 10 μM DCFH-DA (Solarbio, China, D6470) at 37 °C for 15 min. Cells were then washed three times with PBS, and images were observed using a fluorescence microscope.

### Preparation of conditioned medium

Panc-1, MiaPaCa-2, and BxPC-3 cells were seeded into 6-well plates and allowed to reach a confluency of 90%. Subsequently, the cells were treated for 24 h with medium containing either 2.5 μM RP-6306 or without the inhibitor. The medium was then replaced with normal growth medium. After 12 h, the conditioned medium (CM) was collected. The supernatant was filtered through a 0.22 μm filter to remove debris and stored at −80 °C prior to use.

### Macrophage isolation and in vitro polarization

Murine bone marrow-derived macrophages (BMMs) were isolated from the femurs and tibias of 6-week-old C57BL/6 mice. The cells were cultured in Alpha-modified Eagle’s medium (alpha-MEM) supplemented with 10% FBS and 25 ng/ml Recombinant mouse M-CSF (CB34, Novoprotein), with medium changes every two days until the cell density exceeded 90%. For in vitro polarization, BMMs were seeded into 12-well plates and cultured until the cell density reached over 90%. The medium was then replaced with CM in a 1:1 ratio. After 8 h of stimulation, BMMs were harvested for RNA extraction.

### RNA sequencing analysis

MiaPaCa-2 cells were treated with RP-6306 or RP-6306+Gemcitabine or DMSO for 48 h. Then, total RNA was extracted from cells using TRIzol (Invitrogen). Library construction and sequencing were performed by LC-Bio Technology (Hangzhou, China). The average insert size for the final cDNA library was 300 ± 50 bp. At last, we performed the 2 × 150 bp paired-end sequencing (PE150) on an illumina Novaseq™ 6000 (LC-Bio Technology CO., Ltd., Hangzhou, China) following the vendor’s recommended protocol. All primary data in RNA sequencing (RNA-seq) analysis have been uploaded to the Mendeley Data (10.17632/js46crns3j.2).

### Bioinformatics analysis of RNA-seq

Then sequence quality was also verified using fastp. HISAT2 (https://ccb.jhu.edu/software/hisat2) was used to map reads to the reference genome of Homo sapiens GRCh38. The mapped reads of each sample were assembled using StringTie (https://ccb.jhu.edu/software/stringtie) with default parameters. Then, all transcriptomes from all samples were merged to reconstruct a comprehensive transcriptome using gffcompare (https://github.com/gpertea/gffcompare/). After the final transcriptome was generated, StringTie and was used to estimate the expression levels of all transcripts. StringTie was used to perform expression level for mRNAs by calculating FPKM (FPKM = [total_exon_fragments / mapped_reads(millions) × exon_length(kB)]). The differentially expressed mRNAs were selected with fold change > 2 or fold change < 0.5 and with parametric F-test comparing nested linear models (*p* value < 0.05) by R package edgeR (https://bioconductor.org/packages/release/bioc/html/edgeR.html).

### Preparation of cell Membrane-derived vesicles

BxPC-3 cells were harvested using a cell scraper and washed twice with PBS. The collected cells were then resuspended in 1 ml of Membrane Protein Extraction Reagent A containing phenylmethylsulfonyl fluoride (PMSF, 1 mM). After incubation on ice for 15 min, the mixture underwent two cycles of freeze-thaw between liquid nitrogen and room temperature. The mixture was then centrifuged at 700 × *g* for 10 min at 4 °C to remove nuclei and unbroken cells. The supernatant was further centrifuged at 12,000 × *g* for 10 min at 4 °C to obtain the membrane fraction (BxPC-3M). To prepare GEM + RP-6306@BxPC-3M, Gemcitabine, RP-6306, and the membrane fraction were suspended in PBS. The mixture was thoroughly mixed and sonicated for 10 min in an ice bath. The suspension was then extruded using a liposome extruder (Avanti, USA) through polycarbonate filters of 1 μm, 400 nm, and 200 nm (10 cycles each filter). The resulting BxPC-3M and GEM + RP-6306@BxPC-3M were resuspended in PBS and stored at −80 °C.

### Transmission electron microscopy (TEM) for morphological observation of vesicles

The vesicles (BxPC-3M and GEM + RP-6306@BxPC-3M) were diluted in PBS. A drop of each suspension was placed onto a 400-mesh copper grid coated with carbon and air-dried. The samples were then stained with 2% uranyl acetate and images of the TEM grids were acquired using TEM.

### Nanoparticle tracking analysis (NTA)

The hydrodynamic diameter and Zeta potential of the vesicles were measured using a Nanoparticle Tracking Analysis instrument (PARTICLE METRIX, PMX120). After calibrating the instrument with standard particles (100 nm PS beads, polystyrene microspheres), samples diluted 2000-fold in 1× PBS were introduced into the sample chamber. Real-time, dynamic images of the vesicles were observed and recorded through the computer display.

### In vitro uptake of vesicles (cancer cell uptake of GEM + RP-6306@BxPC-3M)

BxPC-3M and GEM + RP-6306@BxPC-3M were labeled with the lipophilic dye DiI for fluorescence tagging. PANC-1, MiaPaCa-2, and BxPC-3 cells were seeded in a 4-well confocal dish and cultured overnight at 37 °C. Subsequently, the cells were co-incubated with the fluorescently labeled GEM + RP-6306@BxPC-3M for 4 h. After fixation with 4% paraformaldehyde, the cell membranes were stained with TRITC Phalloidin (Solarbio, China, CA1610). Fluorescent images of the cells were captured using a confocal laser scanning microscope (CLSM). The entire preparation process was conducted away from strong light to protect the fluorescent dyes.

### In vivo targeting of homologous tumors

Animal experiments were conducted in accordance with the guidelines set by the Animal Care and Use Committee of Sir Run Run Shaw Hospital, Zhejiang University School of Medicin (SRRSH202306130).

Initially, 1 × 10^6 BxPC-3 cells were subcutaneously injected into the left axilla of each nude mouse to establish a tumor-bearing model. Subsequently, the mice were administered intravenous injections of either PBS, solutions containing free GEM + RP-6306, BxPC-3M, or GEM + RP-6306@BxPC-3M. The dosage of Gemcitabine was set at 15 mg/kg per mouse, while the dosage of RP-6306 was 9 μM. 24 h after injection, the mice were anesthetized and subjected to in vivo imaging using the Maestro in vivo imaging system (Cambridge Research & Instrumentation, Inc, Woburn, USA). Following imaging, the mice were euthanized, and the tumors along with major organs (heart, liver, spleen, lungs, and kidneys) were dissected. After rinsing with cold saline, fluorescence images of these tissues were acquired using the Maestro in vivo imaging system.

### In vivo antitumor studies in a BxPC-3 subcutaneous xenograft mouse model

Female BALB/c nude mice, aged 4–5 weeks, were inoculated with BxPC-3 cells in the left axillary region to facilitate the growth of tumor xenografts over approximately three weeks. The in vivo experiments were divided into three phases.

Initially, the mice were randomly assigned into three groups (each consisting of six mice) and subjected to various treatments: PBS, RP-6306 (dosages 2.5 μM and 5 μM). Subsequently, the mice were distributed into four groups and received different treatments: PBS, RP-6306 (dosage 2.5 μM), Gemcitabine (20 mg/kg), or a combination of RP-6306 and Gemcitabine. Gemcitabine was administered once per week, whereas RP-6306 was administered every other day, both via intraperitoneal injection, for a duration of 21 days. Lastly, the mice were again randomly divided into four groups, which received tail vein injections of PBS, GEM + RP-6306, BxPC-3M, and GEM + RP-6306@BxPC-3M. The dosages of Gemcitabine and RP-6306 were 15 mg/kg and 9 μM, respectively, with a frequency of administration once per week.

Tumor size and mouse body weight were measured every three days. Tumor volume was calculated using the formula V = W^2^L/2, where W is the shortest diameter, and L is the longest diameter. At the conclusion of the experiment, all mice were euthanized. The tumors and major organs (heart, liver, spleen, lungs, and kidneys) were then excised, fixed, routinely processed into paraffin, and sectioned at a thickness of 2 µm for histological examination using Hematoxylin and Eosin (H&E) staining.

### Statistical analysis

All data were analyzed using GraphPad Prism version 9.0. Differences between two groups were assessed using the Student’s *t*-test. Differences among three or more groups were evaluated using one-way analysis of variance (ANOVA). All data apart from involving animal were from at least three independent experiments and data were presented as mean ± standard deviation, and a *p*-value of <0.05 was considered statistically significant. The statistical analysis data for the IHC data for tumor tissues are presented as scatterplots, with the bars representing the median values.

## Results

### PKMYT1 is highly expressed in PDAC and is associated with survival prognosis

Protein kinases orchestrate a myriad of vital biological processes, encompassing metabolism, transcription, cell division, motility, and programmed cell death [4]. Within this diverse family, serine/threonine protein kinases, leveraging ATP as a phosphate donor, catalyze the phosphorylation of serine or threonine residues on target proteins [16]. This subset includes cyclin-dependent kinases, mitogen-activated protein kinases (MAPKs), protein kinase D, nattokinase, DNA-dependent protein kinases, Aurora kinases, and prokallikrein, each integral to the development of antitumor therapeutics [4].

**Fig. 1:**
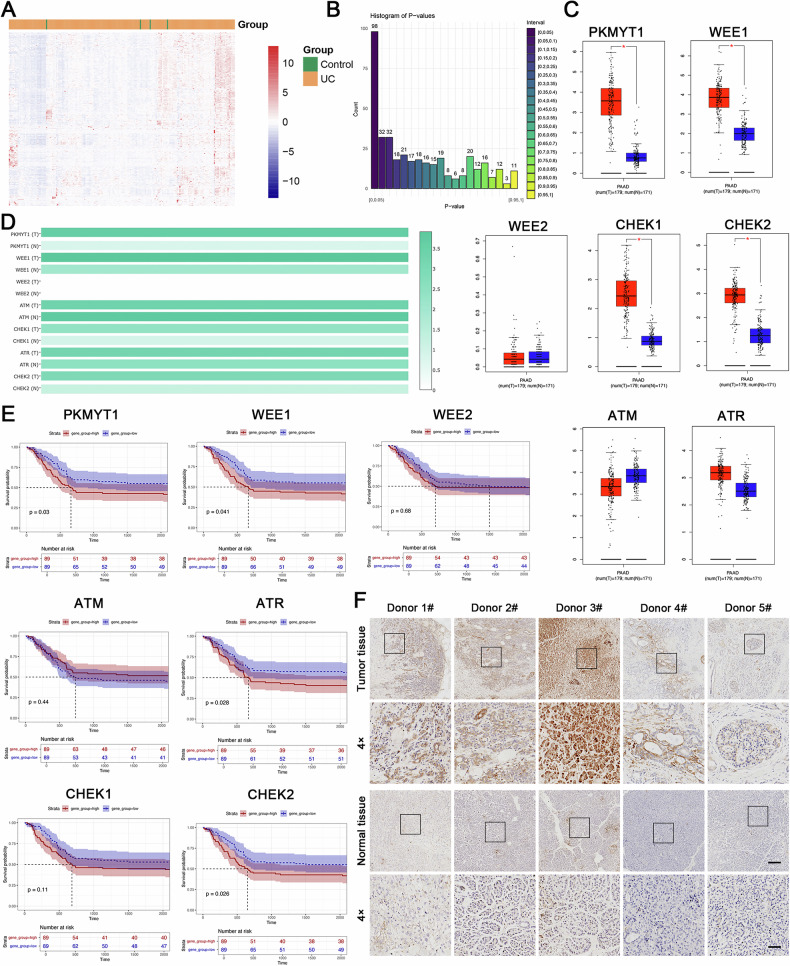
PKMYT1 is highly expressed in PDAC and associated with survival prognosis. **A** Heatmap of serine/threonine kinase family expression in PDAC. **B** Statistical analysis of the correlation between serine/threonine kinase family and survival prognosis in PDAC. **C** Expression of WEE1, WEE2, PKMYT1, ATM, ATR, CHK1, and CHK2 in human pancreatic cancer tissues (*n* = 179) and non-cancerous tissues (*n* = 171) in the TCGA-PAAD (pancreatic adenocarcinoma) cohort. **D** Heatmap of expression levels of WEE1, WEE2, PKMYT1, ATM, ATR, CHK1, and CHK2 in pancreatic cancer tissues and non-cancerous tissues. **E** Kaplan-Meier survival curves for overall survival showing the correlation of WEE1, WEE2, PKMYT1, ATM, ATR, CHK1, and CHK2 with survival prognosis in PDAC patients in the GEO dataset. **F** Immunohistochemical analysis of PKMYT1 in cancerous tissues and adjacent normal pancreatic tissues of PDAC patients. Representative images are shown. Scale bars: 200 μm and 50 μm.

To probe the correlation between the serine/threonine kinase gene family and the pathogenesis of pancreatic adenocarcinoma (PAAD), we conducted an analysis on the expression levels of 399 serine/threonine kinases in pancreatic cancer versus adjacent normal tissues, utilizing data from the TCGA database (Fig. 1A, Table. S1). A proportion of these genes (25.86%) pronounced significant alterations in expression within pancreatic cancer tissues (Fig. 1B).

The role of cell division is paramount in cancer progression, where mitotic proliferation and the precision of chromosome segregation are critical to the cellular lifecycle. Within the serine/threonine kinase family, specific genes such as WEE1, WEE2, PKMYT1, ATM, ATR, CHK1, and CHK2 uniquely regulate the cell cycle (Fig. 1C, D). Notably, PKMYT1 exhibited the most significant expression disparity between tumor and adjacent normal tissues in pancreatic cancer, and survival analyses have substantiated a significant correlation between PKMYT1 expression and the prognosis of pancreatic cancer, underscored by its *P*-value (Fig. 1E). To further elucidate the potential role of PKMYT1 in PDAC, we assessed its expression in human PDAC specimens. Immunohistochemical (IHC) analysis revealed elevated expression of PKMYT1 in PDAC tissues compared to adjacent non-tumor tissues (Fig. 1F).

### RP-6306, a specific inhibitor of PKMYT1, significantly suppresses PDAC in vitro

**Fig. 2:**
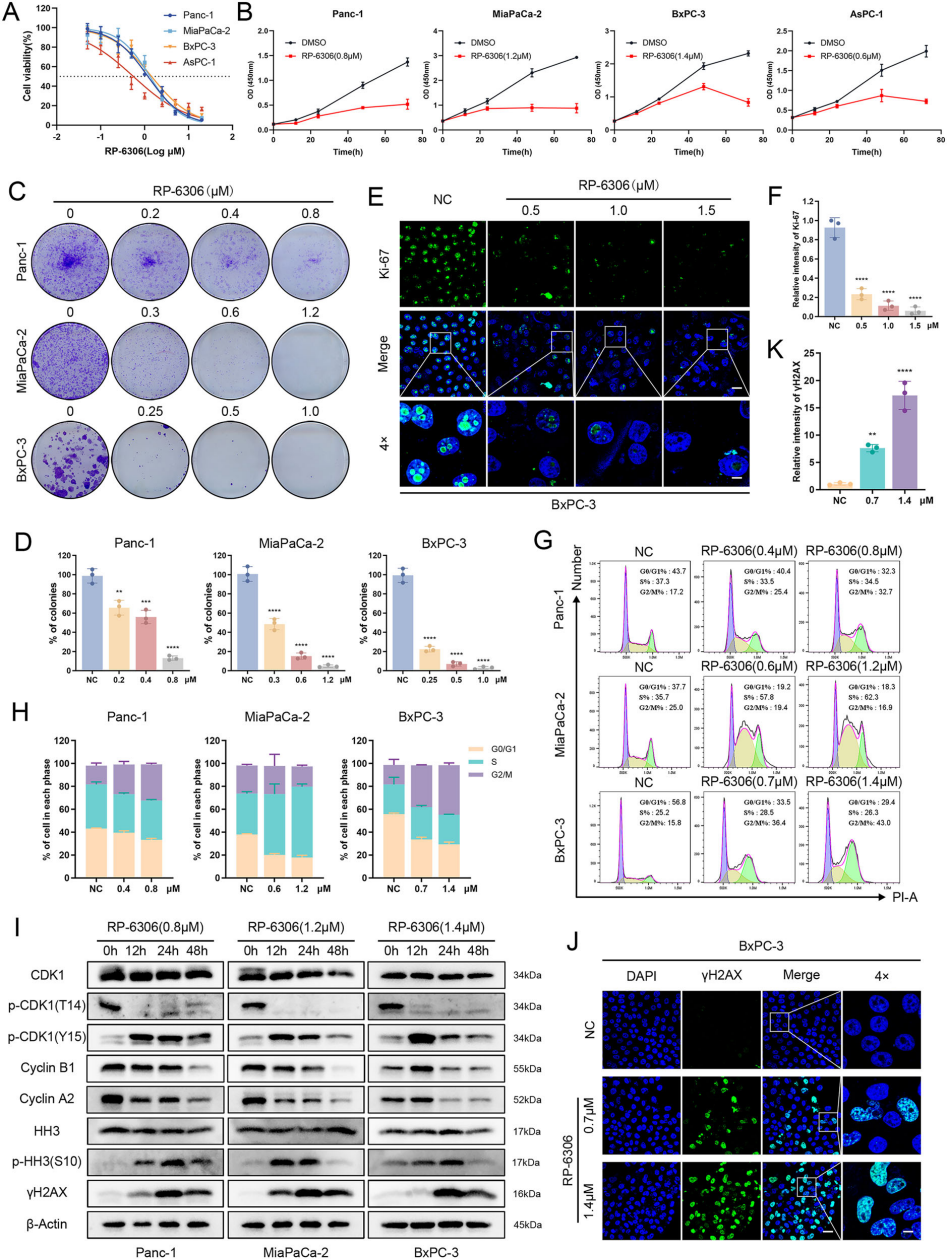
RP-6306 (a PKMYT1 inhibitor) inhibits the proliferation of pancreatic cancer cells in vitro and affects the cell cycle. **A** Cell viability of Panc-1, MiaPaCa-2, BxPC-3, and AsPC-1 cells measured using CCK-8 assay after 48 hours of treatment with RP-6306. **B** Cell viability of Panc-1, MiaPaCa-2, BxPC-3, and AsPC-1 cells at various time points after RP-6306 treatment, assessed via CCK-8 assay. **C** Colony formation assay of pancreatic cancer cells treated with different concentrations of RP-6306 for 72 h followed by culture for 7–10 days without the drug. Remaining cells were fixed and stained with crystal violet. **D** Relative number of colonies formed by pancreatic cancer cells treated with RP-6306. **E** Immunofluorescence staining for Ki-67 in BxPC-3 cells after 48 h of RP-6306 treatment, with nuclei counterstained with DAPI (blue). Scale bars: 100 μm and 25 μm. **F** Relative fluorescence intensity of Ki-67 in BxPC-3 cells. **G** Flow cytometric analysis of the cell cycle to determine changes in the proportions of G0/G1, S, and G2/M phases in pancreatic cancer cells after 48 h of RP-6306 treatment. **H** Distribution of cell cycle phases (G0/G1, S, and G2/M) in pancreatic cancer cells treated with RP-6306. **I** Western blot analysis of pancreatic cancer cells to identify changes in the expression of cell cycle-related proteins after 48 hours of RP-6306 treatment. **J** Immunofluorescence staining for γH2AX in BxPC-3 cells after 48 h of RP-6306 treatment, with nuclei counterstained with DAPI (blue). Scale bars: 100 μm and 25 μm. **K** Relative fluorescence intensity of γH2AX in BxPC-3 cells. The statistical analyses were performed with the ANOVA. Statistical significance is shown in the figure as follows: *p < 0.05; **p < 0.01; ***p < 0.001; or ****p < 0.0001. Each experiment was performed in triplicate, and the error bars represented the mean ± SD.

As a targeted inhibitor of the serine/threonine kinase PKMYT1, we evaluated the effects of RP-6306 on pancreatic cancer cell lines. CCK-8 assays demonstrated that RP-6306 markedly inhibited the growth of Panc-1, MiaPaCa-2, BxPC-3, and AsPC-1 cells, with IC50 values ranging from 0.554 to 1.435 μM over 48 h (Fig. 2A, B). Supplementary validation through colony formation assays and Ki-67 immunofluorescence staining corroborated the robust anti-proliferative efficacy of RP-6306 (Figs. 2C–F, S1A, B). PKMYT1 orchestrates cell cycle regulation by phosphorylating CDK1 on its Thr14 residue, a critical modification that curtails CDK1 activity and impedes its interaction with cyclin B, pivotal for the orchestration of cell cycle progression and mitotic initiation. Our analyses elucidate that RP-6306 instigates a cell cycle arrest, predominantly augmenting the proportion of cells in the G2/M phase and eliciting a conspicuous surge in S phase cells in MiaPaCa-2 (Fig. 2G, H). Concomitantly, RP-6306-mediated dephosphorylation of CDK1 at Thr14 in PDAC cells was observed, accompanied by a discernible downregulation of cyclin A and B (Fig. 2I). Past research posits that a disruption in PKMYT1 kinase activity derails the G2/M checkpoint, precipitating premature mitotic entry and resultant genomic instability [17]. Aligning with these precedents, our findings reveal an escalation in cells transitioning into mitosis, as indicated by heightened p-HH3 (Ser10) levels and an accumulation of DNA damage, as demonstrated by increased γ-H2AX levels (Fig. 2I). Additionally, immunofluorescent staining manifested a significant escalation in pan-nuclear γ-H2AX positive cells, further underscoring the genomic perturbations engendered by RP-6306 (Figs. 2J, K, S1C, D).

### RP-6306 inhibits PDAC via PANoptosis-inflammation

**Fig. 3:**
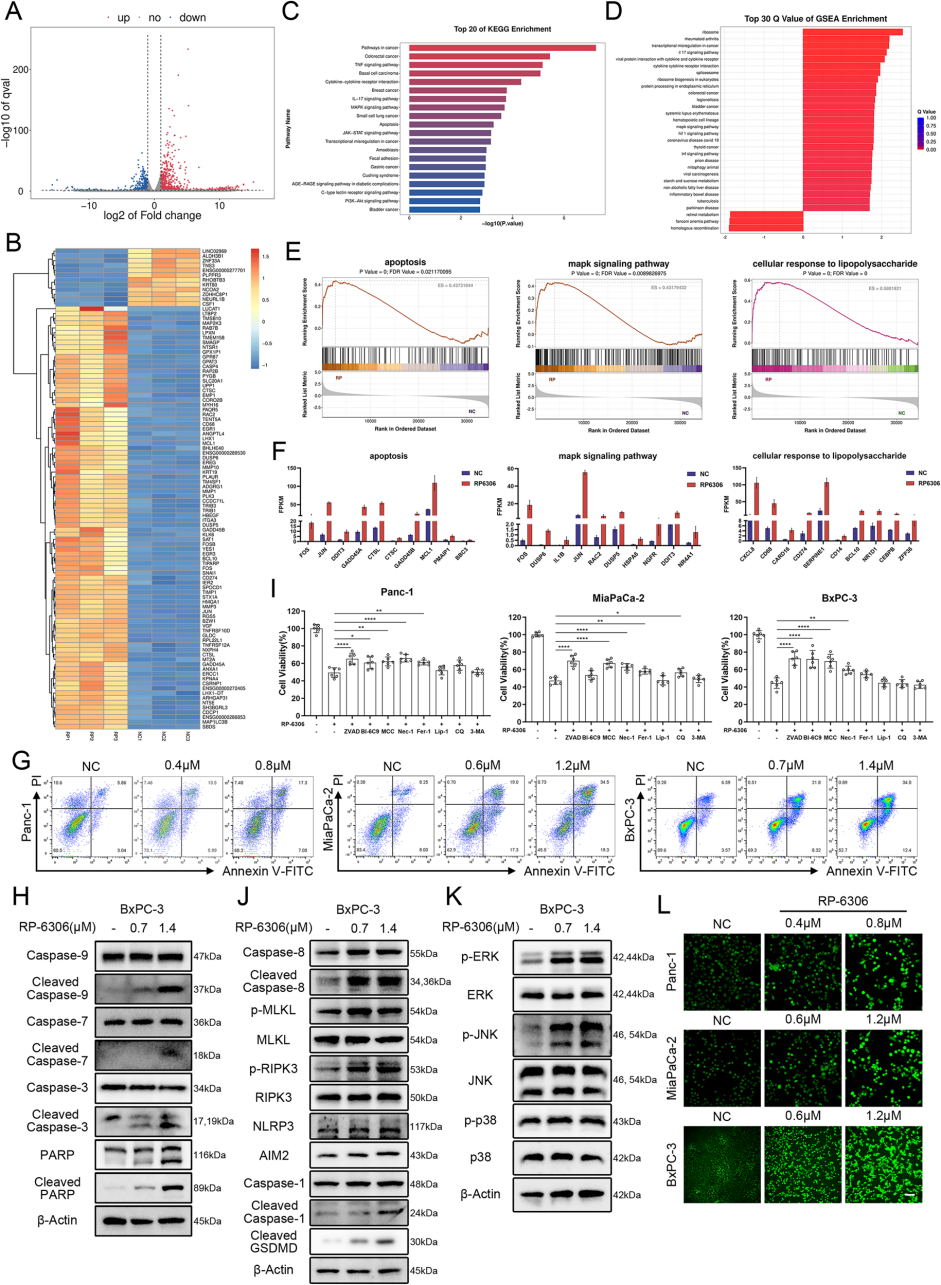
RP-6306 inhibits PDAC through PANoptosis-inflammation pathways. **A** Volcano plot of significantly differentially expressed genes between MiaPaCa-2 cells treated with RP-6306 and the negative control group. Red dots represent upregulated genes; blue dots represent downregulated genes. **B** Heatmap of the top 100 most significantly differentially expressed genes between MiaPaCa-2 cells treated with RP-6306 and the negative control group, with red indicating relative upregulation. **C** KEGG enrichment analysis of significantly differentially expressed genes between MiaPaCa-2 cells treated with RP-6306 and the negative control group. **D** GSEA enrichment analysis based on significantly differentially expressed genes between MiaPaCa-2 cells treated with RP-6306 and the negative control group. **E** Enriched pathways identified in KEGG and GSEA analyses include apoptosis, MAPK, and lipopolysaccharide response. **F** Specific genes significantly upregulated in the enriched pathways identified in D. The statistical analyses were performed with the Student’s *t*-test. **G** Flow cytometric analysis of Annexin V-PE/7-AAD apoptosis assay to determine the proportion of apoptotic pancreatic cancer cells after 48 h of treatment with RP-6306. **H** Western blot analysis of apoptosis-related proteins in BxPC-3 cells after 48 h of RP-6306 treatment. **I** Cell viability of BxPC-3 cells measured using the CCK8 assay after 24 h of treatment with RP-6306 (Panc-1, MiaPaCa-2: 2 μM; BxPC-3: 3 μM) in the presence or absence of Z-VAD-FMK (25 μM), BI-6C9 (10 μM), MCC950 (20 μM), Necrostatin-1 (20 μM), Ferrostatin-1 (10 μM), Liproxstatin-1 (5 μM), Chloroquine (20 μM), 3-Methyladenine (5 mM). The statistical analyses were performed with the ANOVA. **J** Western blot analysis of proteins related to necroptosis and pyroptosis in BxPC-3 cells after 48 h of RP-6306 treatment. **K** Western blot analysis of proteins related to the MAPK pathway in BxPC-3 cells after 48 h of RP-6306 treatment. **L** Immunofluorescence staining for ROS in pancreatic cancer cells after 48 h of RP-6306 treatment. Scale bar: 300 μm. Statistical significance is shown in the figure as follows: *p < 0.05; **p < 0.01; ***p < 0.001; or ****p < 0.0001. Each experiment was performed in triplicate, and the error bars represented the mean ± SD.

To delineate the mechanisms by which RP6306 counteracts pancreatic cancer, we utilized transcriptome sequencing to pinpoint critical transcriptional changes. Upon administering 1.2 μM RP6306 to MiaPaCa-2 cells for 48 h, we discerned 895 genes with significant upregulation and 763 genes with marked downregulation, as depicted in the corresponding volcano plot (Fig. 3A). Subsequent differential analysis of the top 100 genes predominantly accentuated upregulated pathways, as evidenced by heatmap visualization (Fig. 3B). Although RP6306 ostensibly targets PKMYT1, hypothesizing a predominant influence on the core cell cycle during mitotic activities, the KEGG pathway enrichment of differentially expressed genes (DEGs) unexpectedly underscored a substantial engagement in inflammatory activation and responses, including pathways such as TNF, IL-17, MAPK, and JAK-signaling (Fig. 3C).

To obviate potential biases in pathway interpretation arising from DEG analysis, we employed Gene Set Enrichment Analysis (GSEA), which intriguingly continued to underscore these inflammatory pathways among the top 30 upregulated gene sets (Fig. 3D). Significantly, the apoptosis pathway exhibited notable upregulation of specific genes, such as FOS, JUN, DDIT3, GADD45A, and CTSL, implying that RP6306 treatment precipitates an apoptotic cascade (Fig. 3E, F). Moreover, the MAPK signaling pathway, a critical mediator of inflammatory responses, was prominently featured in both differential enrichment analysis and GSEA, culminating in the overexpression of certain critical genes, such as FOS, DUSP6, IL1B, JUN, and RAC2 (Fig. 3E, F). Additionally, the escalated activation of intrinsic inflammatory genes within the cells and the amplified cellular responses to lipopolysaccharide and interleukin 1 underscored an intensified cellular response to external inflammatory stimuli, such as CXCL8, CD68, CARD16, CD274, and SERPINE1 (Fig. 3E, F). Flow cytometric assays further corroborated these findings, demonstrating a marked escalation in both early and late apoptotic populations, alongside a concomitant diminution in cell viability subsequent to RP-6306 exposure (Figs. 3G, S2A). Further molecular investigations revealed that post RP-6306 treatment, there was a pronounced augmentation in the cleavage of effector caspases-3 and -7, with initiator caspases 9 also exhibiting enhanced cleavage (Figs. 3H, S2B, C). However, when we attempted to rescue the cell death induced by RP-6306 treatment using the apoptosis inhibitor Z-VAD-FMK, we observed that it did not fully restore the viability of pancreatic cancer cells affected by RP-6306 (Fig. 3I). Intriguingly, while the necroptosis inhibitor Necrostatin-1, the ferroptosis inhibitor Ferrostatin-1, and the pyroptosis inhibitor MCC950 each partially ameliorated the observed decrement in cell viability induced by RP-6306 (Fig. 3I), none fully reinstated viability to baseline levels observed in untreated controls, hinting at RP-6306’s capability to simultaneously instigate PANoptosis.

Through an extensive review of pertinent literature, we have elucidated a novel inflammatory cell death pathway, designated as PANoptosis, which is precipitated by distinct triggers and modulated by the intricate dynamics of the PANoptosome complex [18]. Among the comprehensively characterized forms of programmed cell death (PCD)—namely pyroptosis, apoptosis, and necroptosis—each pathway is distinctly regulated by complex molecular mechanisms that facilitate the initiation, transduction, and execution of cellular demise. Notably, these pathways exhibit significant intercommunication [9].

Consequently, we further investigated how RP-6306 treatment affects these cell death pathways at the molecular level. Cells subjected to RP-6306 manifested vigorous phosphorylation of the pseudokinase mixed lineage kinase-like domain (MLKL), a critical necroptosis effector (Figs. 3J, S2D), concurrently with increased phosphorylation of receptor-interacting protein kinase 3 (RIPK3), a molecule that not only triggers MLKL phosphorylation but also facilitates the interplay between apoptosis and necroptosis [19]. Caspase-8, a key molecule mediating crosstalk between apoptosis and necroptosis, displayed a significant increase in its cleaved form following treatment with RP-6306 (Figs. 3J, S2D) [20]. Our examination extended to pyroptosis markers, where we observed significant upregulation in the cleavage of gasdermin D (GSDMD), a key executor in pyroptosis known for its role in forming membrane pores under specific cellular stress conditions [21]. GSDMD-induced pyroptosis is characterized by inflammasome signaling (caspase-1-dependent) and noncanonical signaling (caspase-11-dependent) [9]. In this study, we found that RP-6306 enhances the caspase-1 signaling pathway. (Figs. 3J, S2D). This treatment also potentiated the Caspase-1 signaling cascade, further activating the NLRP3 inflammasome pathway, which is typically triggered by toll-like receptor 4 (TLR4) and damage-associated molecular patterns (DAMPs), as well as the AIM2 inflammasome pathway, responsive to mitochondrial (mt) DNA and other forms of double-stranded DNA (Figs. 3J, S2D). In summary, RP-6306 not only facilitates GSDMD cleavage but also robustly activates a network of upstream signaling pathways including NLRP3, AIM2, and Caspase-1 in pancreatic cancer cells (Figs. 3J, S2D). Collectively, these data underscore RP-6306’s capacity to orchestrate PANoptosis, integrating apoptotic, necroptotic, and pyroptotic modalities, leading to decreased cell viability in pancreatic cancer models.

Corroborating transcriptomic sequencing data, RP-6306 administration in pancreatic cancer cells catalyzed the activation of the inflammation-linked MAPK pathway, marked by significant elevations in the phosphorylation levels of ERK, JNK, and p38 (Figs. 3K, S2E). Concurrent investigations have elucidated that mitochondrial impairment augments intracellular ROS, thereby precipitating PANoptosis [22]. Furthermore, our transcriptomic evaluations have disclosed a pronounced enrichment of genes upregulated in response to mitochondrial distress subsequent to RP-6306 treatment. Employing the DCFH-DA assay to assess cellular ROS concentrations, fluorescence microscopy substantiated a notable augmentation in dichlorofluorescein (DCF) fluorescence within RP-6306-treated pancreatic cancer cells (Figs. 3L, S2F). Within the tumor microenvironment, tumor-associated macrophages (TAMs) are instrumental, modulating cancer metastasis, angiogenesis, and immune evasion [23]. Depending on the milieu, TAMs exhibit flexibility in polarization towards either a pro-inflammatory M1 phenotype or an anti-inflammatory M2 phenotype [23]. Pioneering studies have demonstrated that ROS function as an initiating signal that enhances the polarization of macrophages towards the M1 phenotype [24]. Post treatment of pancreatic cancer cells with RP-6306-enriched media, the harvested supernatant was utilized to induce polarization in BMMs. Quantitative PCR analysis revealed that RP-6306 treatment prompted discernible elevations in markers associated with M1 polarization, including CD86, IL-6, IL-1β, iNOS, and TNF-α (Fig. S2G).

### RP-6306 inhibits PDAC growth in vivo

**Fig. 4:**
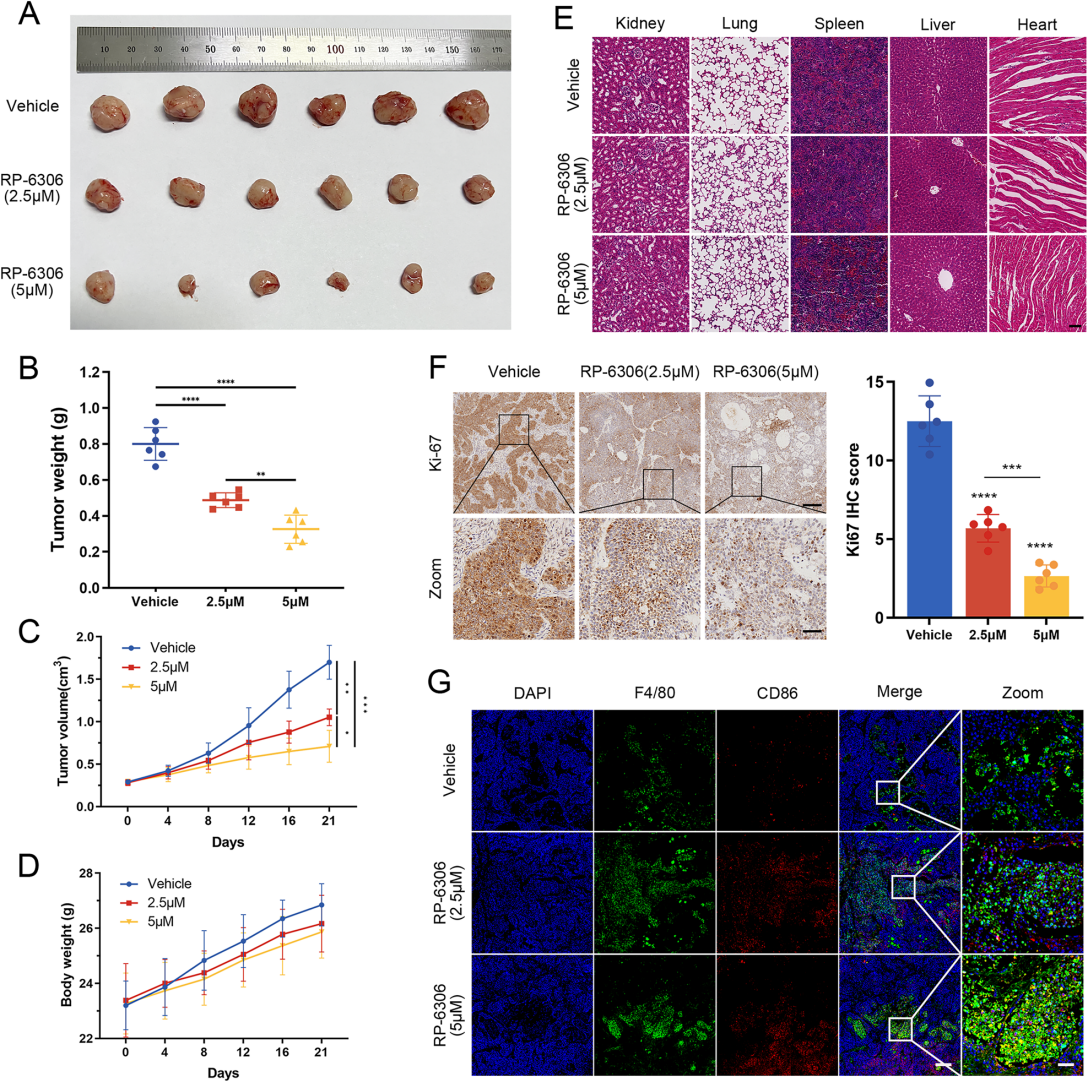
In vivo inhibition of pancreatic cancer cells by RP-6306. **A** Images of subcutaneous tumors in each nude mouse across three groups: vehicle, RP-6306 (2.5 μM), and RP-6306 (5 μM). **B** Tumor weight for each group. Data are presented as scatterplots, with the bars indicating the median value. **C** Growth curves of tumors for each group, and the error bars represented the mean ± SD. **D** Body weight changes in nude mice for each group, and the error bars represented the mean ± SD. **E** Hematoxylin and eosin (H&E) staining of major organs (kidney, lung, spleen, liver, and heart) in each group to assess tissue damage caused by RP-6306. **F** Immunohistochemical (IHC) analysis of Ki-67 expression in tumors from each group and statistical analysis of Ki-67 IHC staining; scale bars: 200 μm and 50 μm. Data are presented as scatterplots, with the bars indicating the median value. **G** Immunofluorescence staining for CD86 in tumors from different groups, with macrophages marked by F4/80 staining, and nuclei counterstained with DAPI (blue); scale bars: 200 μm and 40 μm. The statistical analyses were performed with the ANOVA. Statistical significance is shown in the figure as follows: *p < 0.05; **p < 0.01; ***p < 0.001; or ****p < 0.0001.

To elucidate the suppressive impact of RP-6306 on the in vivo proliferation of BxPC-3 cells, a xenograft tumor model was established utilizing immunodeficient nude mice. Within this framework, the solitary administration of RP-6306 over a 21-day period elicited a substantial diminution in both tumor size and weight, while maintaining the baseline body weight of the mice across the administered dosages (Fig. 4A–D). Histopathological evaluations, employing Hematoxylin and Eosin (H&E) staining, revealed no significant RP-6306-induced organotoxicity in the kidneys, lungs, spleen, liver, or heart (Fig. 4E). Moreover, immunohistochemical analysis unveiled a pronounced reduction in Ki67 expression within pancreatic tumor tissues following treatment with RP-6306 (Fig. 4F), indicative of decreased cellular proliferation. Complementary tissue immunofluorescence assays further demonstrated an upregulation of the M1 macrophage marker CD86 and a concurrent downregulation of the M2 marker CD163 in RP-6306-treated tumor tissues (Figs. 4G, S3A). Cumulatively, these results robustly highlight the formidable antitumor efficacy of RP-6306 against human pancreatic cancer in an in vivo setting.

### RP-6306 enhances the chemosensitization to gemcitabine through the PANoptosis-inflammation pathway

**Fig. 5:**
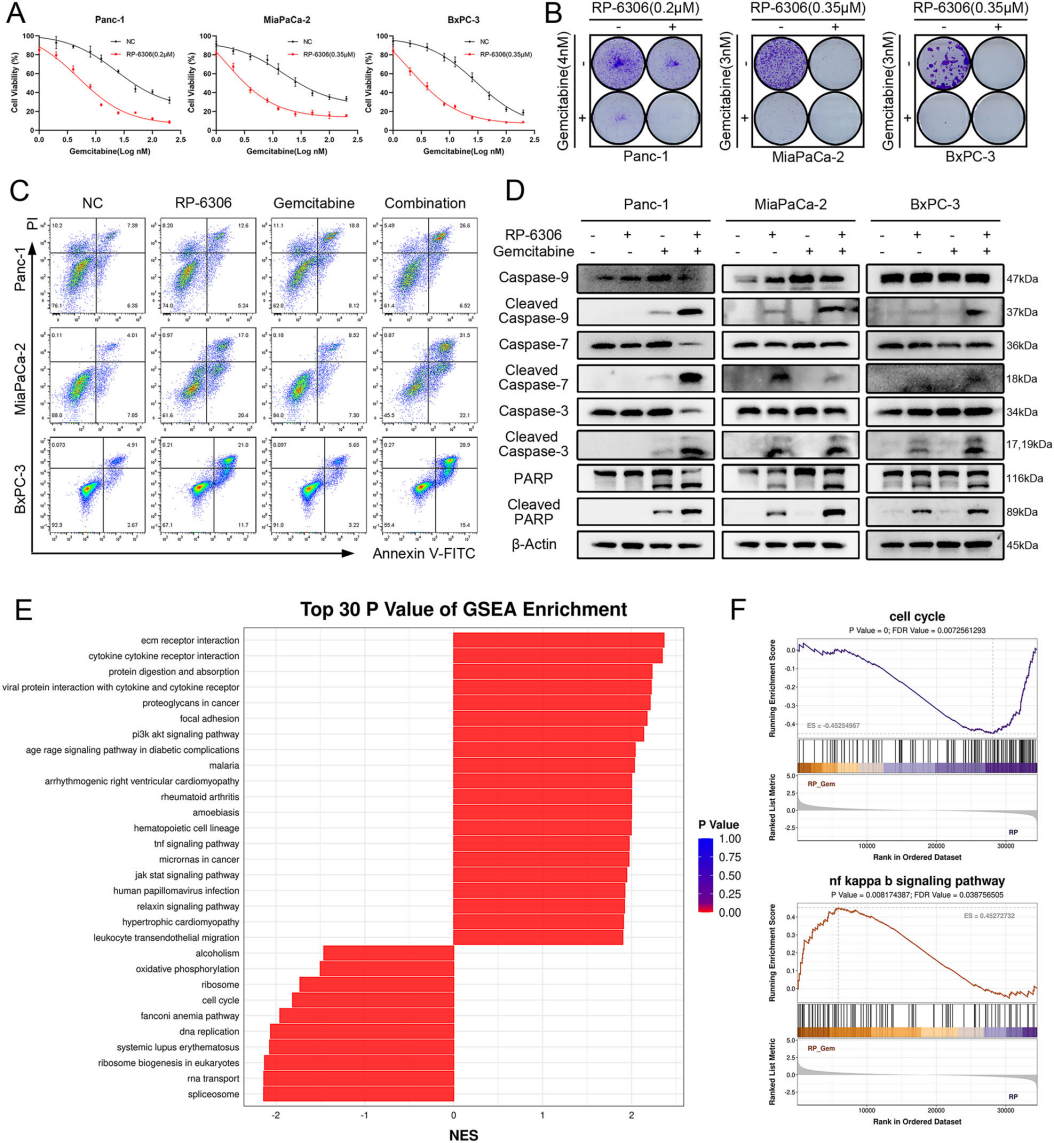
RP-6306 enhances the in vitro response of gemcitabine chemotherapy in pancreatic cancer. **A** Cell viability of Panc-1, MiaPaCa-2, BxPC-3, and AsPC-1 cells was measured using the CCK8 assay after 48 h of treatment with RP-6306 alone or in combination with GEM (gemcitabine). **B** Colony formation assay of pancreatic cancer cells treated with RP-6306 alone or in combination with GEM for 72 h followed by culturing for 7–10 days without drug. Remaining cells were fixed and stained with crystal violet. **C** Flow cytometric analysis of Annexin V-PE/7-AAD apoptosis assay to determine the proportion of apoptotic pancreatic cancer cells after 48 h of treatment with RP-6306 alone or in combination with GEM. **D** Western blot analysis of apoptosis-related protein expression in pancreatic cancer cells after 48 h of treatment with RP-6306 alone or in combination with GEM. **E** Gene Set Enrichment Analysis (GSEA) based on significantly differentially expressed genes between the RP-6306 + GEM combination group and the RP-6306 alone group in MiaPaCa-2 cells. **F** Enriched pathways identified in the GSEA include cell cycle, and NF-kappa-B signaling pathway. The statistical analyses were performed with the ANOVA. Statistical significance is shown in the figure as follows: *p < 0.05; **p < 0.01; ***p < 0.001; or ****p < 0.0001. Each experiment was performed in triplicate, and the error bars represented the mean ± SD.

Gemcitabine, a pivotal chemotherapeutic agent for pancreatic cancer, primarily manifests its cytotoxic effects by inducing DNA damage, inhibiting DNA synthesis, and promoting cellular apoptosis [25]. Nevertheless, the emergence of chemoresistance, exacerbated by the activation of DNA repair pathways, significantly undermines its therapeutic potential by reducing tumor susceptibility to the drug [26]. In an endeavor to evaluate the synergistic interaction between the PKMYT1 inhibitor and gemcitabine in curtailing the proliferation of PDAC, we employed RP-6306, a PKMYT1 inhibitor, in established PDAC cell lines. The inhibitor was administered at a sub-threshold concentration (IC20), which curtails cellular proliferation by less than 20%, and was used in conjunction with a titrated range of gemcitabine concentrations over a 48 h period. Our findings revealed that while gemcitabine alone diminished PDAC cell viability in a dose-responsive manner, the co-administration of RP-6306 and gemcitabine synergistically enhanced this effect, yielding a more significant reduction in cell viability than was observed with gemcitabine monotherapy (Fig. 5A). Colony formation assays corroborated these results, showing a markedly reduced colony count in the combination therapy group compared to groups treated with either RP-6306 or gemcitabine alone (Figs. 5B, S4A). Flow cytometry analysis reinforced these findings, indicating that the combination therapy notably outperformed monotherapy in inducing apoptotic cell death, as demonstrated by heightened levels of apoptosis markers (Figs. 5C, S3B). Furthermore, Western blot analysis of apoptosis-related proteins, including cleaved-PARP, cleaved-caspase 3, cleaved-caspase 7, and cleaved-caspase 9, revealed that the expression levels of these proteins were consistently higher in the combination therapy group, underscoring the enhanced apoptotic response elicited by the synergistic interaction of RP-6306 and gemcitabine in pancreatic cancer cells (Fig. 5D). Gemcitabine alone has limited efficacy and does not improve quality of life. In stark contrast to the mitotic storm induced by RP6306, gemcitabine’s primary metabolites are incorporated into DNA, predominantly disrupting the G1/S phase transition and precipitating mitotic aberrations. The antagonistic interactions between these compounds in tumor suppression have sparked considerable interest, leading to an in-depth investigation of their synergistic anti-tumor effects through combined usage. To elucidate the underlying mechanisms, transcriptome sequencing has been strategically utilized.

GSEA reveals that, compared to the exclusive use of RP6306, co-administration with gemcitabine markedly suppresses pathways pivotal to DNA replication and cell cycle progression, alongside significant downregulation of specific genes (Figs. 5E, F, S3C). This evidence robustly supports gemcitabine’s profound antagonistic influence on cell cycle regulation relative to RP6306. Intriguingly, gemcitabine administration further augments inflammatory signaling pathways; beyond the pronounced upregulation of the TNF and MAPK pathways observed with RP6306 alone, there is also enhanced expression of genes within the NF-kappa-B signaling pathway, suggesting an intensified induction of PANoptosis (Figs. 5E, F, S3C). These findings underscore PANoptosis as a potentially vital mechanism for tumor cell elimination and a promising target for cell cycle-focused anticancer therapeutics.

### RP-6306 and gemcitabine synergistically inhibit the growth of pancreatic cancer cells in vivo

**Fig. 6:**
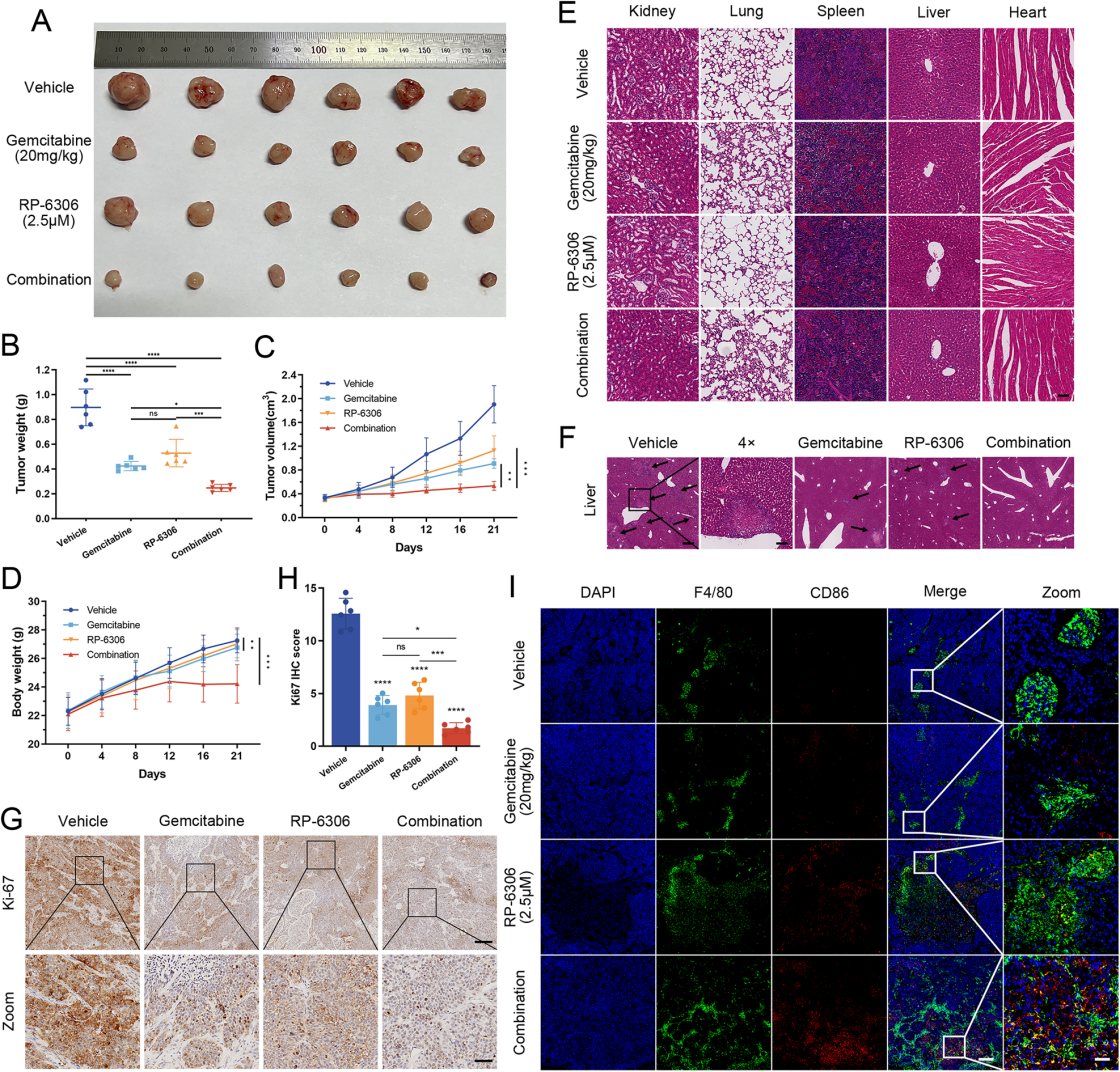
In vivo inhibition of PDAC tumor growth by the combination of RP-6306 and gemcitabine chemotherapy. **A** Images of subcutaneous tumors in each nude mouse across four groups: vehicle, gemcitabine (20 mg/kg), RP-6306 (2.5 μM), and combination. **B** Tumor weight for each group. Data are presented as scatterplots, with the bars indicating the median value. **C** Growth curves of tumors for each group, and the error bars represented the mean ± SD. **D** Body weight changes in nude mice for each group, and the error bars represented the mean ± SD. **E** Hematoxylin and eosin (H&E) staining of major organs (kidney, lung, spleen, liver, and heart) in each group to assess tissue damage from RP-6306 and gemcitabine, either alone or in combination; scale bar: 400 μm. **F** H&E staining illustrating pancreatic cancer metastatic foci in the liver of each group of nude mice; scale bars: 400 μm and 100 μm. **G** Immunohistochemical (IHC) analysis of Ki-67 expression in tumors from each group; scale bars: 200 μm and 50 μm. **H** Statistical analysis of Ki-67 IHC staining. Data are presented as scatterplots, with the bars indicating the median value. **I** Immunofluorescence staining for CD86 in tumors from different groups, with macrophages marked by F4/80 staining, and nuclei counterstained with DAPI (blue); scale bars: 200 μm and 40 μm. The statistical analyses were performed with the ANOVA. Statistical significance is shown in the figure as follows: *p < 0.05; **p < 0.01; ***p < 0.001; or ****p < 0.0001.

To rigorously evaluate the potential of RP-6306 to augment the antitumor activity of gemcitabine in vivo, BXPC-3 PDAC xenograft models were employed. Mice harboring tumors received intraperitoneal injections of RP-6306 (2.5 μM) bi-daily, gemcitabine (20 mg/kg) on a weekly basis, or a synergistic regimen combining both agents for a three-week period. Tumor volumes and body weights were systematically recorded at four-day intervals. Data illustrated in Fig. 6A–C reveal that RP-6306 alone curtailed tumor proliferation by 41.09%, whereas gemcitabine monotherapy facilitated a reduction of 52.77% in tumor mass. Notably, the concomitant administration of both compounds achieved a superior tumor suppression rate of 72.39%. A marked decrement in body mass was observed in the cohort receiving combination therapy, relative to the control group, as delineated in Fig. 6D. Histopathological examination of H&E-stained specimens disclosed incidences of glomerulonephritis and pneumonia in mice treated with gemcitabine, either as a single agent or in combination, though other organs remained largely unaffected (Fig. 6E). Analysis of liver sections stained with H&E indicated the presence of pancreatic cancer metastases; specifically, metastatic foci were identified in three out of six control mice, one mouse in each of the RP-6306 and gemcitabine monotherapy groups, and none in the combination therapy cohort (Fig. 6F). Immunohistochemical assessments demonstrated a pronounced reduction in the proliferation marker Ki67 in neoplasms subjected to the combined treatment regimen (Fig. 6G, H). Furthermore, tissue immunofluorescence staining underscored the treatment’s efficacy in upregulating the M1 macrophage marker CD86 and downregulating the M2 macrophage marker CD163 within the tumor milieu (Figs. 6I, S3B). Collectively, these findings substantiate that RP-6306 significantly potentiates the in vivo antitumor efficacy of gemcitabine.

### The preparation and characterization of cell membrane vesicles derived from PDAC (BxPC-3M), along with their in vitro properties

In the pursuit of highly specific targeting within the realm of cancer therapy and diagnostics, the ultimate objective remains the selective engagement of cancerous cells. In our investigation, while the combined application of RP-6306 and gemcitabine yielded a significant suppression of pancreatic cancer, an observable decline in the body weight of the murine models pointed to a potential cytotoxicity associated with the treatment regimen. Concurrently, we explored the utility of cell-derived vesicles as emergent nanocarriers designed to potentiate antitumor immunity within the ambit of immunotherapeutic strategies [11].

**Fig. 7:**
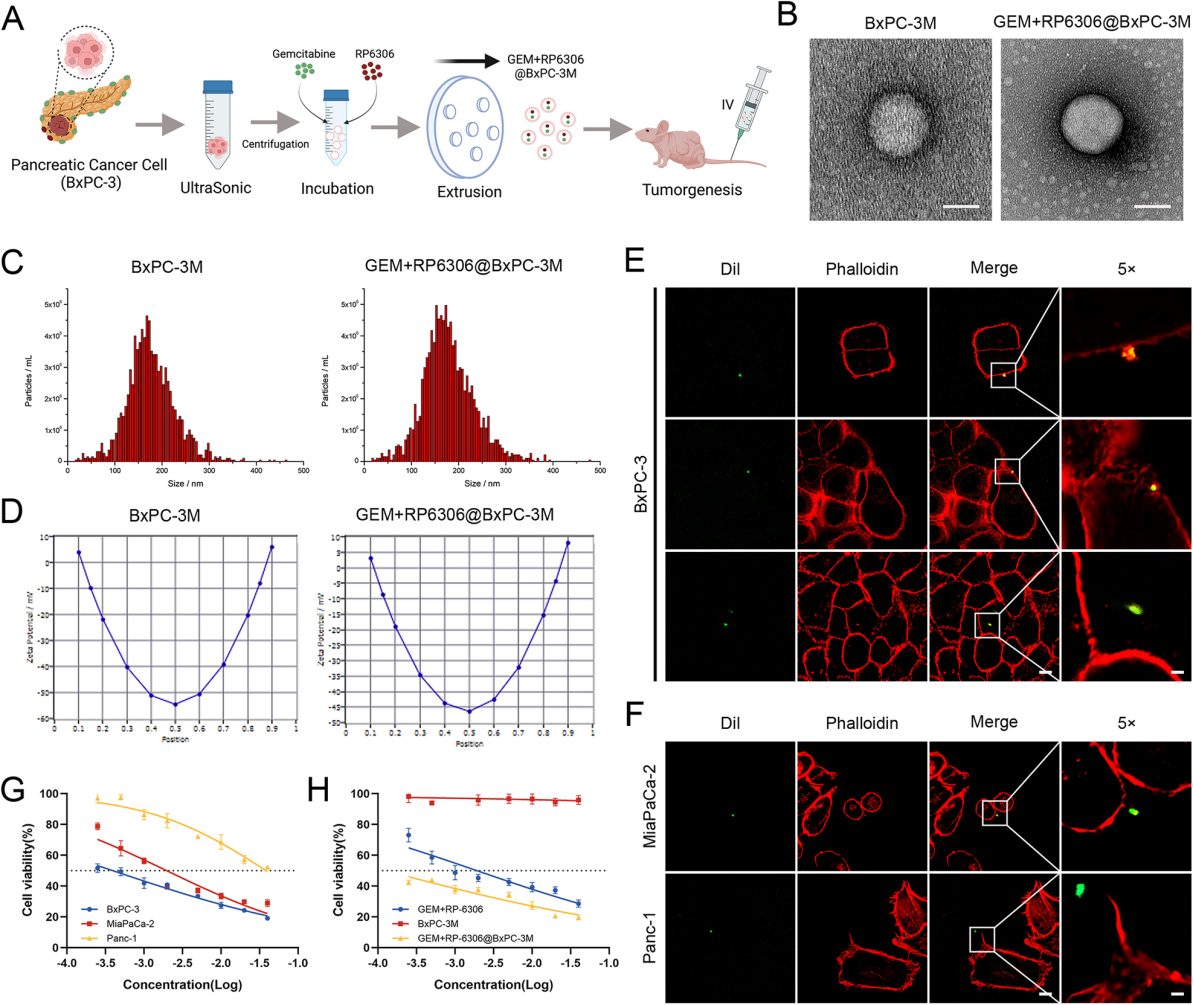
Preparation and characterization of PDAC-derived cell membrane vesicles (BxPC-3M) and their properties in vitro. **A** Schematic illustration of the preparation of nanoscale vesicles derived from the pancreatic cancer cell membrane of BxPC-3, loaded with drugs for personalized cancer therapy. **B** TEM images of BxPC-3M and GEM + RP-6306@BxPC-3M. **C** Size distribution of BxPC-3M and GEM + RP-6306@BxPC-3M. **D** Zeta potential measurements of BxPC-3M and GEM + RP-6306@BxPC-3M. **E** Representative CLSM images of BxPC-3 cells incubated with DiI-labeled GEM + RP-6306@BxPC-3M for 4 h. Scale bars: 10 μm and 2 μm. **F** Representative CLSM images of Panc-1 and MiaPaCa-2 cells incubated with DiI-labeled GEM + RP-6306@BxPC-3M for 4 hours. Scale bars: 10 μm and 2 μm. **G** Cell viability of Panc-1, MiaPaCa-2, and BxPC-3 cells measured using the CCK8 assay after 48 h of treatment with GEM + RP-6306@BxPC-3M. Each experiment was performed in triplicate, and error bars are shown as the mean ± SD. **H** Cell viability of BxPC-3 cells measured using the CCK8 assay after 48 hours of treatment with BxPC-3M, free GEM + RP-6306, and GEM + RP-6306@BxPC-3M. Each experiment was performed in triplicate, and error bars are shown as the mean ± SD.

To this end, we fabricated vesicles derived from the membrane of the BxPC-3 pancreatic cancer cell line, designated as BxPC-3M, and subsequently encapsulated the therapeutic agents to form GEM + RP-6306@BxPC-3M (Fig. 7A). Confirmation of the vesicular structures was attained via TEM, revealing that the extracellular vesicles (EVs) of BxPC-3M and those encapsulating the drugs measured approximately 50.0 nm and 87.5 nm, respectively, aligning with the established EV dimension range of 50–200 nm (Fig. 7B). Further characterization through nanoparticle tracking analysis (NTA) determined that the average hydrodynamic diameters of BxPC-3M and GEM + RP-6306@BxPC-3M were approximately 168.7 nm and 169.7 nm, respectively, substantiating the successful isolation of the EVs (Fig. 7C). Dynamic light scattering (DLS) analysis elucidated the zeta potentials of BxPC-3M at −9.40 ± 0.52 mV, and GEM + RP-6306@BxPC-3M at −6.89 ± 0.56 mV, thereby maintaining the intrinsic electrical properties of the vesicles (Fig. 7D).

Upon verifying the encapsulation efficacy of gemcitabine and RP-6306, we embarked on evaluating the in vitro homotypic targeting prowess of GEM + RP-6306@BxPC-3M. This was accomplished by co-incubating Dil-labeled GEM + RP-6306@BxPC-3M with various pancreatic cancer cell lines—Panc-1, MiaPaCa-2, and BxPC-3—for a duration of four hours, with subsequent observations using CLSM. Notably, intense green fluorescence was detected within the BxPC-3 cells post-incubation, whereas such fluorescence was absent in Panc-1 and MiaPaCa-2 cells (Fig. 7E, F). This selective cellular uptake prompted further investigations through a short-term administration assay, demonstrating that the cytotoxic efficacy of GEM + RP-6306@BxPC-3M on BxPC-3 cells surpassed that on Panc-1 and MiaPaCa-2, attributable to the homotypic targeting facilitated by the BxPC-3 cell membrane (Fig. 7G). However, GEM + RP-6306@BxPC-3M also exhibited significant cytotoxicity against MiaPaCa-2 cells. This could be attributed to the presence of identical surface adhesion molecules, such as N-cadherin and galectin-3, on the surfaces of different pancreatic cancer cell lines [12]. To further investigate this, we conducted a CCK-8 assay under shorter time conditions (3 h and 6 h) with GEM + RP-6306@BxPC-3M treatment, and the results showed that the cytotoxicity of GEM + RP-6306@BxPC-3M against the parental BxPC-3 cells was significantly higher than that against Panc-1 and MiaPaCa-2 cells (Fig. S5A, B). Additionally, a cytotoxicity assay employing the CCK-8 methodology on BxPC-3 cells indicated that BxPC-3M exhibited commendable biocompatibility, displaying negligible cytotoxicity even at elevated concentrations, whereas GEM + RP-6306@BxPC-3M manifested pronounced cytotoxic effects following the release of the encapsulated drugs, significantly outperforming the equivalent dosage of the free drug combination (Fig. 7H). These findings underscore the high degree of specific self-recognition and affinity of GEM + RP-6306@BxPC-3M for its source cells, heralding a promising avenue for targeted cancer therapy.

### GEM + RP-6306@BxPC-3M demonstrates efficient targeted therapy for PDAC in vivo

**Fig. 8:**
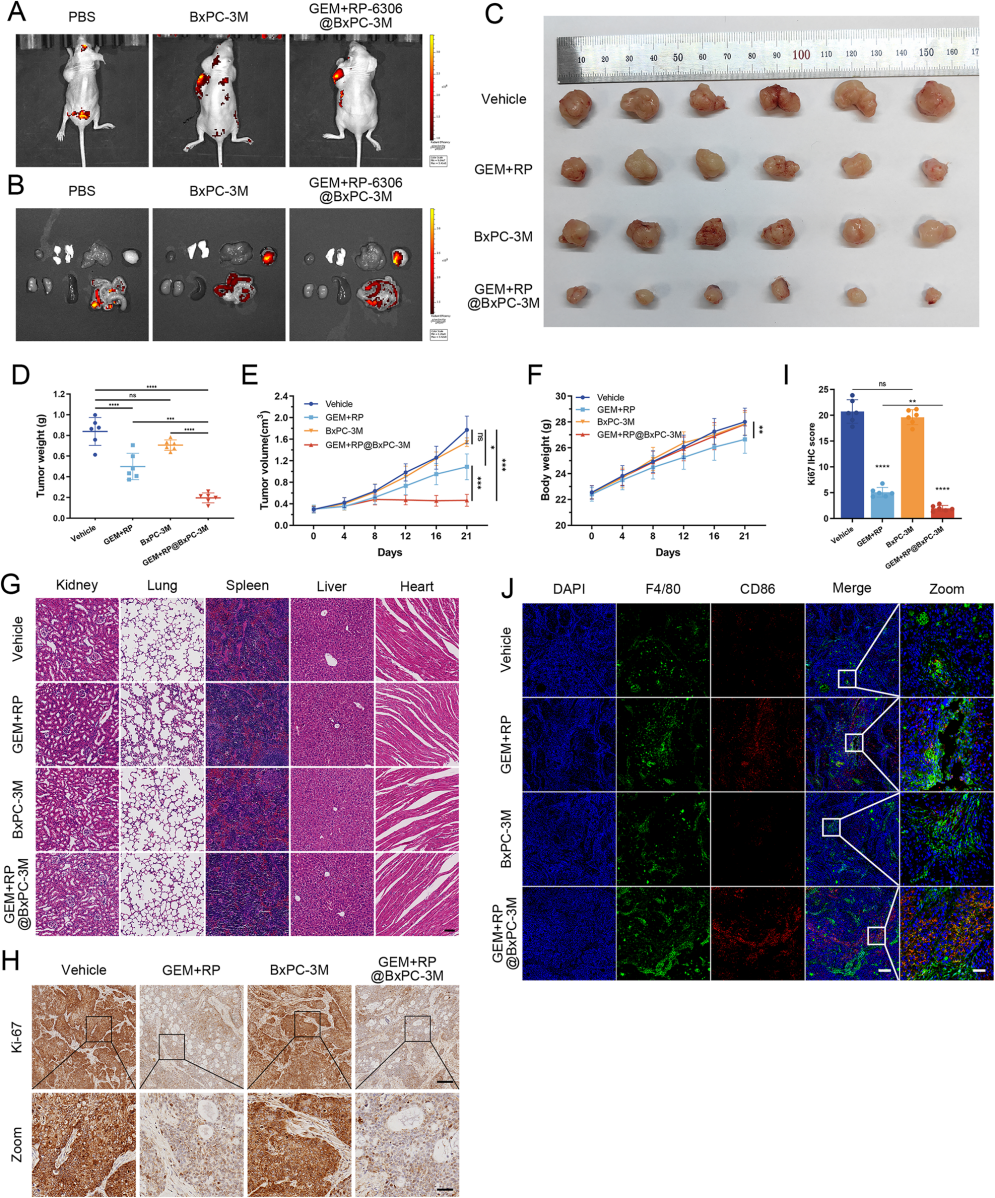
Efficient targeted therapy of PDAC in vivo using GEM + RP-6306@BxPC-3M. **A** Representative in vivo fluorescence images of nude mice bearing subcutaneous tumors 24 h after intravenous injection of BxPC-3M or GEM + RP-6306@BxPC-3M. **B** Representative ex vivo fluorescence images showing the distribution of BxPC-3M and GEM + RP-6306@BxPC-3M in tumors and major organs 24 h post-injection. **C** Images of subcutaneous tumors in each nude mouse across four groups: vehicle, GEM + RP-6306, BxPC-3M, and GEM + RP-6306@BxPC-3M. **D** Tumor weight for each group. Data are presented as scatterplots, with the bars indicating the median value. **E** Growth curves of tumors for each group, and the error bars represented the mean ± SD. **F** Body weight changes in nude mice for each group, and the error bars represented the mean ± SD. **G** Hematoxylin and eosin (H&E) staining of major organs (kidney, lung, spleen, liver, and heart) in each group to assess tissue damage from GEM + RP-6306, BxPC-3M, and GEM + RP-6306@BxPC-3M; scale bar: 400 μm. **H** Immunohistochemical (IHC) analysis of Ki-67 expression in tumors from each group; scale bars: 200 μm and 50 μm. **I** Statistical analysis of Ki-67 IHC staining. Data are presented as scatterplots, with the bars indicating the median value. **J** Immunofluorescence staining for CD86 in tumors from different groups, with macrophages marked by F4/80 staining, and nuclei counterstained with DAPI (blue); scale bars: 200 μm and 40 μm. The statistical analyses were performed with the ANOVA. Statistical significance is shown in the figure as follows: *p < 0.05; **p < 0.01; ***p < 0.001; or ****p < 0.0001.

Buoyed by the auspicious in vitro outcomes of homotypic self-recognition, our investigation advanced toward the in vivo appraisal of tumor self-targeting (TST) attributes of our formulations. Initially, a subcutaneous xenograft model employing BxPC-3 cells was established to mimic PDAC. To probe the in vivo homotypic targeting efficacy of GEM + RP-6306@BxPC-3M, mice harboring uniformly sized tumors on their upper left limbs were randomly selected and administered intravenous injections of either PBS or Dil-labeled BxPC-3M and GEM + RP-6306@BxPC-3M. Twenty-four hours post-injection, imaging revealed pronounced fluorescence at the tumor locales in mice injected with the labeled formulations, contrasting sharply with the minimal intratumoral fluorescence observed in the control cohort, thereby underscoring successful targeting of CDX sites (Fig. 8A). Subsequent ex vivo imaging of principal organs and tumors manifested augmented fluorescence within the tumors, indicating significant tumor accumulation of BxPC-3M and GEM + RP-6306@BxPC-3M (Fig. 8B).

To assess the in vivo antitumor potency of GEM + RP-6306@BxPC-3M, mice bearing BxPC-3 xenografts were segregated into four groups, each receiving weekly intravenous injections of either PBS, free GEM + RP-6306, BxPC-3M, or GEM + RP-6306@BxPC-3M over a span of three weeks. Tumor volume and body weight were diligently monitored at four-day intervals. The visual and quantitative data disclosed vigorous tumor proliferation in the PBS-only cohort (Fig. 8C–E), whereas a substantial mitigation of tumor growth was observed in mice treated with GEM + RP-6306@BxPC-3M. Although the combination of RP-6306 and GEM was associated with discernible side effects, as evidenced by weight loss, the encapsulated drug formulation did not substantially impair the overall health of the mice, suggesting commendable biocompatibility of this personalized cancer therapy platform (Fig. 8F). Histopathological evaluations using H&E staining highlighted instances of glomerulonephritis and pneumonia in the group receiving the free drug combination, yet no notable organ damage was detected in the GEM + RP-6306@BxPC-3M cohort (Fig. 8G). Immunohistochemical assays revealed notably diminished Ki67 staining in tumors treated with GEM + RP-6306@BxPC-3M, indicative of suppressed cellular proliferation (Fig. 8H, I). Furthermore, tissue immunofluorescence staining evidenced a marked elevation in the expression of the M1 macrophage marker CD86 in tumor tissues treated with GEM + RP-6306@BxPC-3M (Fig. 8J), corroborating the robust antitumor efficacy of GEM + RP-6306@BxPC-3M in the CDX model with minimal systemic toxicity.

## Discussion

PDAC is predominantly diagnosed at an advanced clinical stage, invariably linked with dismal prognoses and substantial morbidity [27]. Despite the significant survival advantage conferred by surgical resection, the preponderance of patients are diagnosed with metastatic spread, precluding them from surgical options [27]. Irrespective of eligibility for surgery, the vast majority of patients, committed to therapeutic intervention, are subjected to broad-spectrum chemotherapy [28]. Since the 1990s, therapies centered around gemcitabine, either as monotherapy or in combination regimens, have constituted the principal treatment modality for most individuals afflicted with this malignancy. Regrettably, the emergence of chemoresistance has led to prevalent therapeutic failures among these patients. This persistent issue of gemcitabine resistance poses a formidable challenge within clinical oncology, compelling the development of innovative therapeutic strategies aimed at amplifying the effectiveness of gemcitabine-based treatments or reinstating drug responsiveness in patients with gemcitabine-resistant PDAC.

Encompassing 518 distinct entities, the protein kinase family is instrumental in modulating protein function, thereby exerting a profound influence across virtually all domains of cellular biology [29]. The dysregulation of kinase activity is a critical factor in the etiology of a spectrum of diseases, including autoimmune, cardiovascular, inflammatory, and neurological disorders, in addition to a vast array of cancers [30]. Contemporary research underscores the indispensable role of kinases in gene expression, DNA repair, and the architectural transformation of the tumor microenvironment, thus positioning them as quintessential targets for therapeutic intervention in the current century [31]. Predominantly composed of serine/threonine kinases, the family includes tyrosine kinases solely within the TK and TKL subfamilies. With the majority of FDA-approved kinase inhibitors presently targeting tyrosine kinases, there emerges a significant therapeutic frontier for the development of inhibitors aimed at serine/threonine kinases [32]. Our investigative efforts are centered on serine/threonine kinases, with a particular focus on those associated with the G2/M checkpoint, such as CDK1, PLK1, ATM, ATR, CHK1/2, and the WEE family kinases. Targeted inhibition of CHK1/2 and WEE kinases has the potential to dismantle the G2/M checkpoint, disrupt cell cycle control, and precipitate the accumulation of mitotic DNA damage, culminating in mitotic catastrophe [33–35]. While substantial research and clinical trials are ongoing for inhibitors of ATM, ATR, and CHK1/2, our focus has pivoted towards the lesser-studied WEE kinase family [36]. The WEE kinase family is comprised of merely three members: WEE1, WEE1B, and PKMYT1, with WEE1B being uniquely expressed in germ cells and not yet implicated in oncogenesis [37]. The development of inhibitors targeting WEE1 is challenged by issues such as potential off-target effects and resistance mechanisms [38]. In contrast, PKMYT1 displays a distinct substrate specificity towards CDK1 and is associated with enhanced metastatic potential and diminished overall survival in colorectal and breast cancers, highlighting its role as a potential prognostic biomarker [5, 39, 40]. Importantly, emerging studies indicate that inhibitors of PKMYT1 selectively engage cells experiencing high replicative stress, such as those exhibiting CCNE1 amplification, while inflicting minimal harm on healthy cells [8]. Thus, PKMYT1 is posited as a pivotal driver of tumor aggressiveness, and targeting this kinase presents a promising avenue for the development of novel cancer therapeutics.

Historically, mitotic catastrophe was acknowledged as a meticulously regulated antineoplastic mechanism that impedes the proliferation or survival of neoplastic cells failing to complete mitosis [41]. Originally associated with mitotic arrest, the therapeutic potential of this phenomenon has increasingly been recognized [41]. Irrespective of the specific targets or mechanisms of action of various anticancer agents, they are capable of precipitating mitotic catastrophe following the onset of mitotic arrest [42]. Our findings, corroborated by figures (Figs. 2I–K, S1C, D), indicate that either the silencing of PKMYT1 or administration of RP-6306 disrupts the G2/M checkpoint integrity in pancreatic cancer cell lines, leading to untimely and unregulated entry into mitosis coupled with an accumulation of unresolved DNA damage, culminating in mitotic catastrophe [43]. Contemporary research posits mitotic catastrophe as a distinct cellular demise modality, amalgamating certain morphological and biochemical characteristics of apoptosis and necroptosis [44, 45]. A study has revealed the role of proteasome inhibitors in inducing mitotic pyroptosis, characterizing it as a novel form of cell death associated with mitotic catastrophe [46]. Transcriptomic analyses following RP-6306 treatment in pancreatic cancer cells have revealed a significant upregulation of genes implicated in several inflammation-associated pathways, including MAPK, PI3K/Akt, JAK/Stat, and NF-κB. PANoptosis, a variant of inflammatory cell death, is delineated by a sophisticated regulatory network of multiple signaling molecules and has been linked to infections, inflammation, and oncogenesis [18]. Preclinical models have associated PANoptosis with sterile inflammatory conditions, exemplified by the prevention of chronic recurrent multifocal osteomyelitis in mice through the concurrent deficiency of Caspase-1, Caspase-8, and RIPK3—key PANoptosis components [47]. Consequently, we hypothesize that PANoptosis may manifest as a lethal outcome during mitotic catastrophe. Our experimental data demonstrate that inhibitors targeting various cell death pathways, such as Z-VAD-FMK, necrostatin-1, Ferrostatin-1, and MCC950, only partially ameliorate the compromised viability of pancreatic cancer cells induced by RP-6306 (Fig. 3G). Western blot analyses further validate that RP-6306 treatment substantially elevates the levels of molecules pivotal to apoptosis, necroptosis, and pyroptosis in pancreatic cancer cells (Figs. 3H, J, S2B–D). ROS are recognized as critical markers of PANoptosis, and studies by Lin et al. [22] have demonstrated that NFS1 deficiency, when combined with oxaliplatin, synergistically escalates intracellular ROS levels, thereby triggering PANoptosis. Additionally, research by M. Cornago et al. [48] suggests that HDAC inhibitors elevate ROS production, potentially facilitating DNA damage. Our investigations confirm that RP-6306 treatment markedly increases ROS accumulation in pancreatic cancer cells (Figs. 3L, S2F), substantiating the hypothesis that PANoptosis serves as a terminal event in RP-6306-induced mitotic catastrophe.

The quintessential modalities of PCD—apoptosis, autophagy, ferroptosis, and pyroptosis—encompass distinct mechanistic underpinnings and physiological outcomes [10]. Specifically, pyroptosis is marked by pronounced cellular swelling followed by membrane rupture, facilitating the release of intracellular constituents that recruit inflammatory cells and instigate immune activations [49]. The role of inflammation in tumorigenesis is dual, promoting or suppressing oncogenic processes contingent on its temporal dynamics—acute or chronic [50]. Acute inflammatory responses, when promptly activated, amplify cytotoxic lymphocyte functionalities, thereby precipitating the eradication of malignancies through immune-mediated mechanisms [51]. The orchestration of anti-tumor immune responses by PCD incorporates pyroptosis, ferroptosis, and autophagy, each capable of instigating tumor cell demise and eliciting acute inflammatory cascades [52, 53]. Particularly, M1-type TAMs secrete a repertoire of pro-inflammatory cytokines including IL-1β, IL-6, and tumor necrosis factor-alpha (TNF-α), alongside generating ROS that potentially inflict DNA damage, thus catalyzing innate immune responses conducive to the obliteration of tumor cells. In contrast, M2-type macrophages not only perpetuate tumor progression via chemokine secretion but also manifest treatment resistance through mechanisms such as angiogenesis, immune suppression, and matrix remodeling [54, 55]. Consequently, strategic inhibition of M2 macrophages and fostering their repolarization to the M1 phenotype emerges as pivotal in the realm of cancer immunotherapy. This study’s RT-qPCR analysis elucidates that RP-6306 augments the expression of M1 polarization markers CD86, IL-6, IL-1β, iNOS, and TNF-α in macrophages (Fig. S2G). Complementary in vivo assays substantiate that RP-6306, either as monotherapy or in synergy with gemcitabine, significantly escalates CD86 levels while diminishing CD163 expression in tumor matrices (Figs. 4G, 6I, S3A, B). Furthermore, Western blot findings reveal that RP-6306 treatment activates the inflammatory MAPK pathway within pancreatic cancer cells, evidenced by elevated phosphorylation of p38 MAPK, JNK, and ERK (Fig. 3L). Therefore, we posit that the PANoptosis triggered by RP-6306 may substantially modulate the tumor microenvironment, thereby contributing to the efficacy of tumor immunotherapeutic strategies.

In the realm of oncological therapeutics for pancreatic cancer, regimens predicated on gemcitabine, either as monotherapy or in combination, continue to serve as the cornerstone of chemotherapeutic interventions. Gemcitabine mediates its anti-proliferative effects through direct or indirect disruption of DNA synthesis and replication within neoplastic cells, thereby curtailing their propagation. Nonetheless, this compound also adversely affects normal cellular populations, engendering systemic toxicities. The present investigation elucidates that while the co-administration of RP-6306 and gemcitabine manifests robust synergistic anti-neoplastic properties and mitigates metastatic progression in pancreatic malignancies, it concurrently precipitates adverse phenotypes such as weight reduction and histopathological perturbations—glomerulonephritis and pneumonia specifically—verified through hematoxylin and eosin staining of principal organs (Fig. 6D, E), which are emblematic of gemcitabine-induced cytotoxicity. Moreover, pancreatic cancer’s pronounced resistance to gemcitabine substantially circumscribes its therapeutic utility. The tumor microenvironment (TME) not only fosters the proliferation and dissemination of pancreatic cancer cells but also catalyzes resistance to chemotherapeutic agents [56]. To surmount the formidable challenges of drug delivery within the TME, particularly to zones of resistance that are pivotal in cancer treatment, sophisticated nanotechnologies endowed with intelligent design features have been deployed [57]. Conventional nanocarriers such as liposomes, micelles, polymer nanoparticles, and metallic nanoparticles encounter obstacles including clearance by the reticuloendothelial system and immune surveillance, culminating in severe hepatotoxicity and inadequate drug deposition at the intended sites [58]. In contrast, vesicles derived from cellular membranes have garnered recognition in the arena of biomimetic drug delivery, owing to their retention of the native membrane properties and functionalities of their source cells, which confer enhanced biocompatibility, reduced immunogenicity, minimal toxicity, extended circulatory longevity, and intrinsic targeting aptitudes [12]. In this study, we harnessed vesicles extracted from the BxPC-3 adenocarcinoma cell line, designated BxPC-3M, to encapsulate RP-6306 and gemcitabine. Our findings reveal that GEM + RP-6306@BxPC-3M proficiently targets homologous cells while maintaining commendable biocompatibility and devoid of eliciting cytotoxic effects (Fig. 7E–H). Subsequent in vivo assessments corroborated that BxPC-3M and GEM + RP-6306@BxPC-3M efficiently home to analogous tumor sites, ensuring effective drug accumulation within the neoplastic lesions (Fig. 8A, B). Furthermore, GEM + RP-6306@BxPC-3M demonstrated superior anti-tumor efficacy in CDX models relative to unencapsulated drug treatments, without inducing organ damage, thereby highlighting its potentiated homologous targeting capability. In summation, vesicles derived from natural cell membranes present unprecedented benefits over synthetic nanomaterials and their evolution into a novel delivery system paradigm portends significant advancements in anti-tumor therapeutics.

Although our research indicates that RP-6306 treatment in pancreatic cancer leads to mitotic catastrophe, ultimately triggering PANoptosis, the relationship between mitotic catastrophe and PANoptosis still requires further experimental validation. In mouse experiments, although we observed that the combination of RP-6306 and gemcitabine appears to have some effect on tumor metastasis, the inhibitory effect of the drug on tumor metastasis needs to be further confirmed with an orthotopic tumor model. Additionally, our study shows that treatment with RP-6306 alone or in combination with gemcitabine leads to an upregulation of M1 macrophages and a downregulation of M2 macrophages in the tumor environment, yet further establishment of an NSG mouse immune reconstitution model is required to analyze macrophage polarization in the tumor tissue.

In conclusion, PKMYT1, markedly upregulated in pancreatic cancer, functions as an oncogenic promoter of tumorigenesis. The inhibitor RP-6306 is pivotal in triggering extensive apoptosis across mitotic cells, potentially heralding a novel paradigm of cell death linked to mitotic catastrophe. Concomitantly, the synergistic combination of RP-6306 and gemcitabine enhances antitumor efficacy in the management of pancreatic cancer. Furthermore, vesicles derived from oncogenic cell membranes, utilized as a drug delivery vector, exhibit superior biocompatibility, minimal toxicity, and intrinsic targeting properties, warranting additional investigation for their potential application in oncological therapies.

## Supplementary information

Reproducibility checklist

Supplemental material

Table S1

Original western blots

## Data Availability

The datasets used and/or analyzed during the current study are available from the corresponding author on reasonable request.
