# Supplementary material for: Targeted delivery of the PKMYT1 inhibitor RP-6306 mediates PANoptosis in pancreatic cancer via mitotic catastrophe
Source: Cell Death Dis. 2025 Jul 15;16(1):526. doi: 10.1038/s41419-025-07835-2 (PMC12263950; doi:10.1038/s41419-025-07835-2)
Supplement: Supplementary file 2 — Supplemental material [file 41419_2025_7835_MOESM2_ESM.docx]

**Supplemental material**


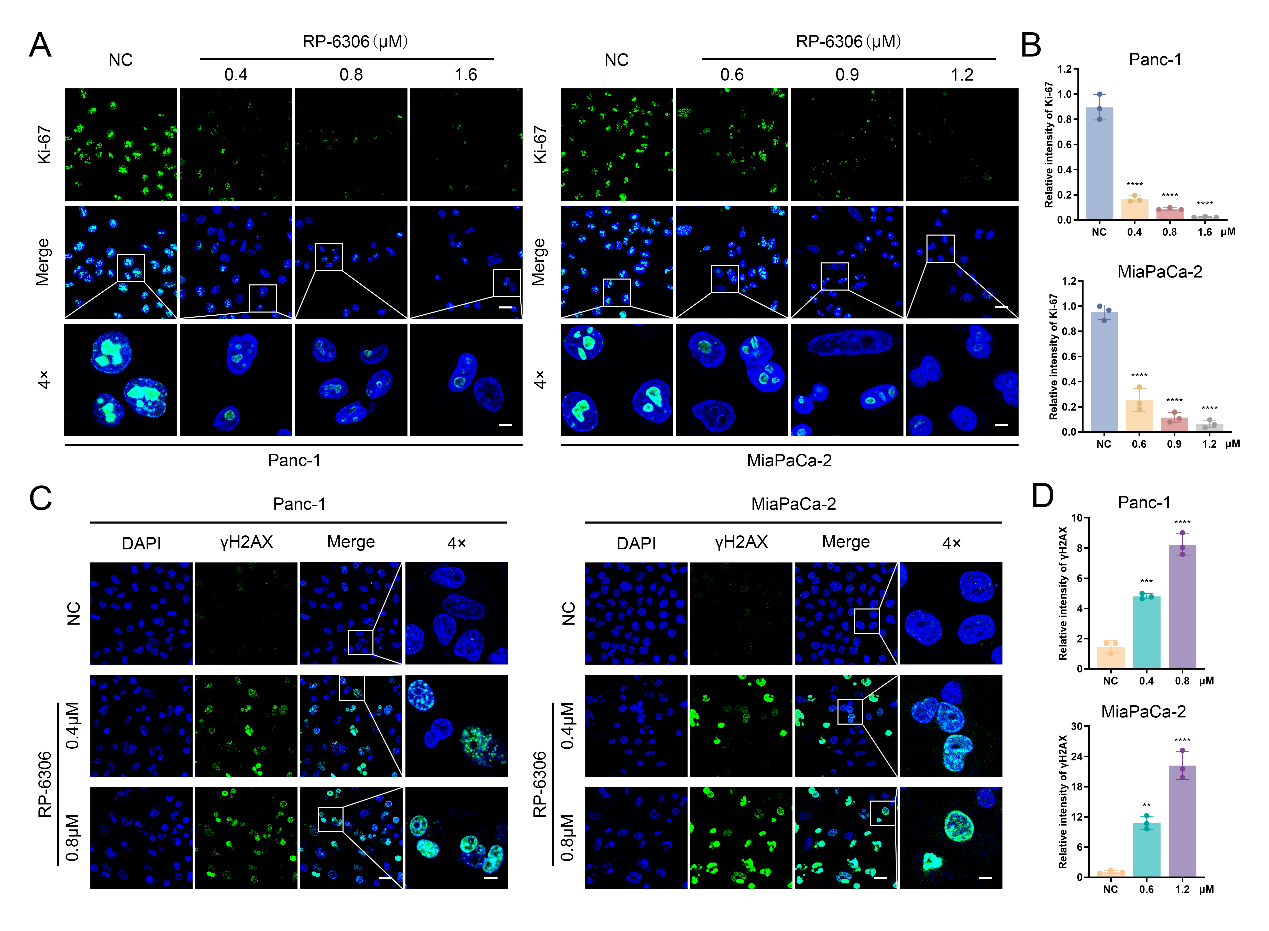


**Figure S1.** Supplementary to Figure 2

(A) Immunofluorescence staining of Ki-67 in Panc-1 and MiaPaCa-2 cells 48 hours after RP-6306 treatment, with nuclei counterstained with DAPI (blue). Scale bars: 100μm and 25μm. (B) Relative fluorescence intensity of Ki-67 in Panc-1 and MiaPaCa-2 cells. (C) Immunofluorescence staining of γH2AX in Panc-1 and MiaPaCa-2 cells 48 hours after RP-6306 treatment, with nuclei counterstained with DAPI (blue). Scale bars: 100μm and 25μm. (D). Relative fluorescence intensity of γH2AX in Panc-1 and MiaPaCa-2 cells. The statistical analyses were performed with the ANOVA. Statistical significance is shown in the figure as follows: *p < 0.05; **p < 0.01; ***p < 0.001; or ****p < 0.0001. Each experiment was performed in triplicate, and the error bars represented the mean ± SD.


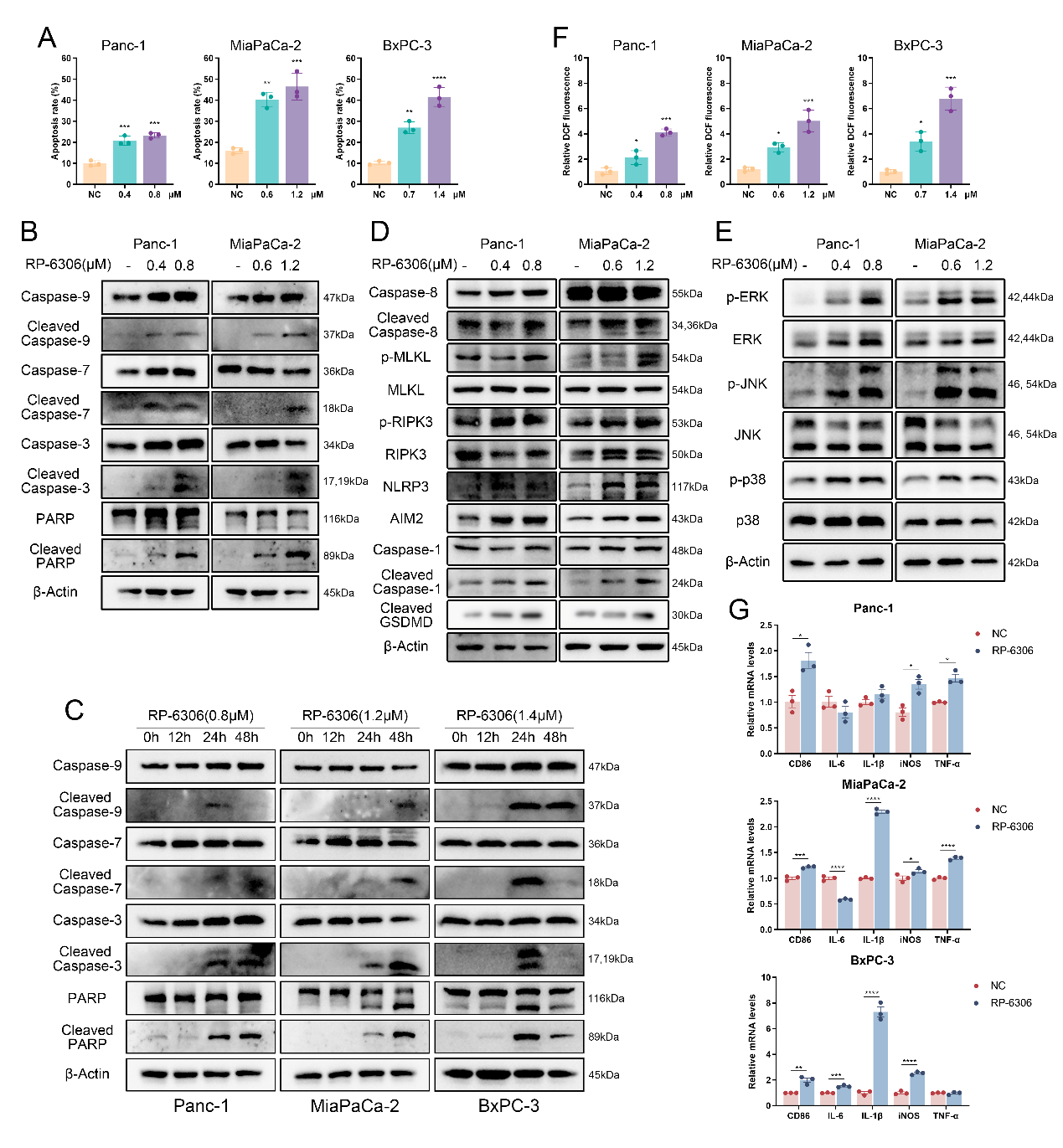


**Figure S2.** Supplementary to Figure 3

(A) Proportion of apoptotic cells in pancreatic cancer cells treated with RP-6306 as shown in Fig. 3H. The statistical analyses were performed with the ANOVA. (B) Western blot analysis of apoptotic protein expression changes in Panc-1 and MiaPaCa-2 cells 48 hours after treatment with RP-6306. (C) Western blot analysis to determine expression changes of apoptosis-related proteins in pancreatic cancer cells after treatment with RP-6306 at various time points and concentrations. (D) Western blot analysis of necroptosis and ferroptosis-related protein expression changes in Panc-1 and MiaPaCa-2 cells 48 hours after RP-6306 treatment. (E) Western blot analysis of MAPK pathway-related protein expression changes in Panc-1 and MiaPaCa-2 cells 48 hours after RP-6306 treatment. (F) Relative fluorescence intensity of ROS in pancreatic cancer cells 48 hours after RP-6306 treatment as shown in Fig. 3L. The statistical analyses were performed with the ANOVA. (G) Conditioned medium (CM) produced by pancreatic cancer cells 24 hours after RP-6306 treatment induces macrophage polarization; RT-qPCR detection of M1-related markers in macrophages. The statistical analyses were performed with the Student's t-test. Statistical significance is shown in the figure as follows: *p < 0.05; **p < 0.01; ***p < 0.001; or ****p < 0.0001. Each experiment was performed in triplicate, and the error bars represented the mean ± SD.


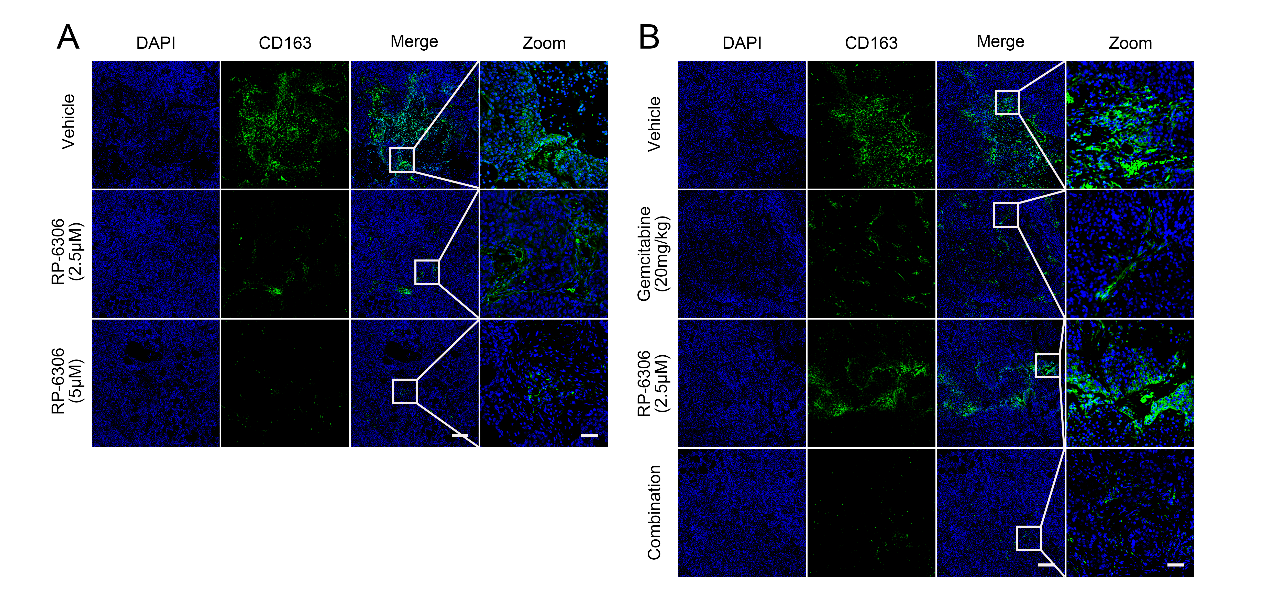


**Figure S3.** Supplementary to Figure 4 and Figure 6

(A) Immunofluorescence staining of CD206 in tumors treated with vehicle, RP-6306 (2.5μM), and RP-6306 (5μM), with nuclei counterstained with DAPI (blue). Scale bars: 200μm and 40μm. (B) Immunofluorescence staining of CD206 in tumors treated with vehicle, Gemcitabine (20mg/kg), RP-6306 (2.5μM), and a combination of treatments, with nuclei counterstained with DAPI (blue). Scale bars: 200μm and 40μm.


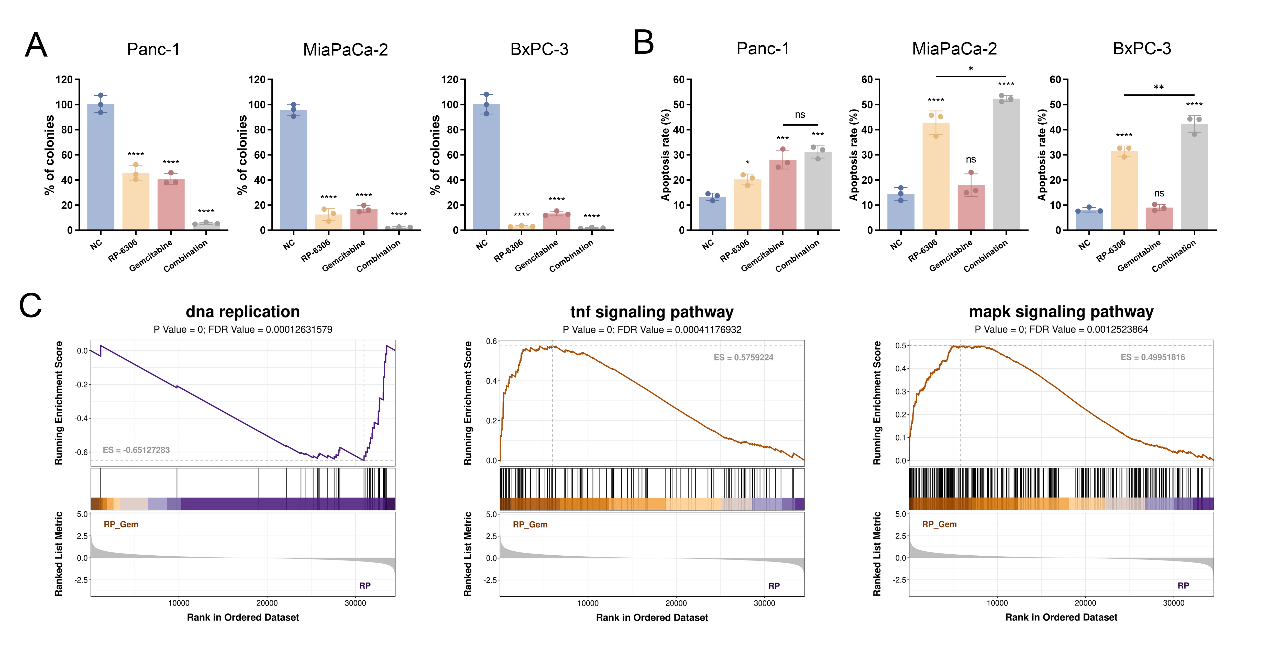


**Figure S4.** Supplementary to Figure 5

(A) Relative number of colonies formed by pancreatic cancer cells treated with RP-6306 and GEM alone or in combination as shown in Fig 5B. (B) Proportion of apoptotic cells in pancreatic cancer cells treated with RP-6306 and GEM alone or in combination as shown in Fig. 5C. (C) GSEA analysis of enriched pathways including DNA replication, TNF, and MAPK signaling pathway. Data presented as mean ± standard deviation. *P<0.05, **P<0.01, ***P<0.001, ****P<0.0001. The statistical analyses were performed with the ANOVA. Statistical significance is shown in the figure as follows: *p < 0.05; **p < 0.01; ***p < 0.001; or ****p < 0.0001. Each experiment was performed in triplicate, and the error bars represented the mean ± SD.

**
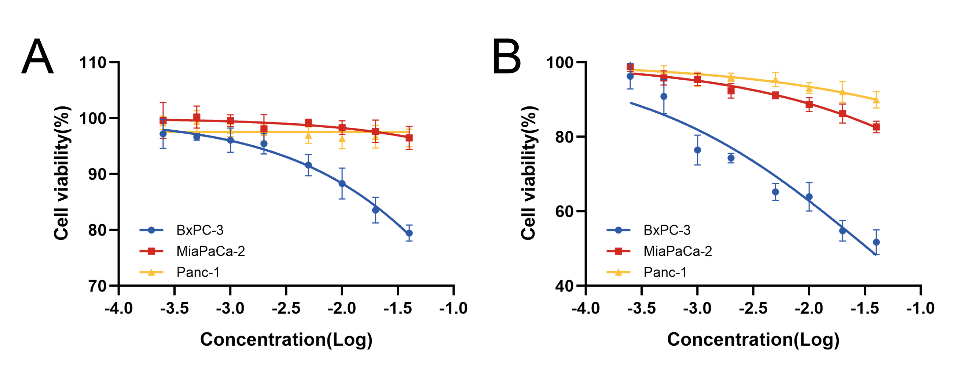
**

**Figure S5.** Supplementary to Figure 7

(A) Cell viability of Panc-1, MiaPaCa-2, and BxPC-3 cells measured using the CCK8 assay after 3 hours of treatment with GEM+RP-6306@BxPC-3M. (B) Cell viability of Panc-1, MiaPaCa-2, and BxPC-3 cells measured using the CCK8 assay after 6 hours of treatment with GEM+RP-6306@BxPC-3M. Each experiment was performed in triplicate, and error bars are shown as the mean ± SD.

**Table S2.** The antibodies used in this article

| Antigen | Brand | Catalog |
| --- | --- | --- |
| Anti-PKMYT1 | proteintech | 67806-1-Ig |
| Anti-Ki67 | Abcam | ab16667 |
| Anti-γH2AX | Abcam | ab26350 |
| Anti-CDK1 | Abcam | ab133327 |
| Anti-p-CDK1 (T14) | CST | #2543S |
| Anti-p-CDK1 (Y15) | CST | #4539 |
| Anti-Cyclin B1 | CST | #12231 |
| Anti-Cyclin A2 | Abcam | ab181591 |
| Anti-Histone H3 | Abways | CY6587 |
| Anti-p-Histone H3 (S10) | CST | #3377P |
| Anti-β-Actin | Abbkine | ABL1010 |
| Anti-Caspase-8 | HUABIO | ET1603-16 |
| Anti-Caspase-9 | CST | #9508 |
| Anti-Cleaved Caspase-9 | CST | #52873 |
| Anti-Caspase-7 | CST | #12827 |
| Anti-Cleaved Caspase-7 | CST | #8438 |
| Anti-Caspase-3 | CST | #14220 |
| Anti-Cleaved Caspase-3 | CST | #9664 |
| Anti-PARP | CST | #9542 |
| Anti-Cleaved PARP | CST | #5625 |
| Anti-p-MLKL | HUABIO | ET1705-51 |
| Anti-MLKL | HUABIO | ET1601-25 |
| Anti-p-RIPK3 | HUABIO | HA721428 |
| Anti-RIPK3 | HUABIO | HA722182 |
| Anti-NLRP3 | proteintech | 19771-1-AP |
| Anti-AIM2 | CST | #63660 |
| Anti-Caspase-1 | Abclonal | A0964 |
| Anti-Cleaved Caspase-1 | CST | #4199 |
| Anti-Cleaved GSDMD | CST | #36425 |
| Anti-p-ERK | CST | #4370 |
| Anti-ERK | CST | #4695S |
| Anti-p-JNK | CST | #9255S |
| Anti-JNK | Abcam | ab179461 |
| Anti-p-p38 MAPK | CST | #9211 |
| Anti-p38 MAPK | Abcam | ab170099 |
| Anti-CD86 | proteintech | 13395-1-AP |
| Anti-CD163 | Abcam | ab182422 |
| Anti-F4/80 | Santa Cruz | sc-377009 |

**Table S3.** qPCR primers

| Gene | Sequence |
| --- | --- |
| Ms-iNOS-F | GGTGAAGGGACTGAGCTGTTA |
| Ms-iNOS-R | TGAAGAGAAACTTCCAGGGGC |
| Ms-CD86-F | GCAGCACGGACTTGAACAAC |
| Ms-CD86-R | TTGTAAATGGGCACGGCAGA |
| Ms-IL-6-F | TCCTACCCCAATTTCCAATGCT |
| Ms-IL-6-R | TGGTCTTGGTCCTTAGCCAC |
| Ms-IL-1β-F | TGCCACCTTTTGACAGTGATG |
| Ms-IL-1β-R | ATGTGCTGCTGCGAGATTTG |
| Ms-TNF-α-F | ATGGCCTCCCTCTCATCAGT |
| Ms-TNF-α-R | TTTGCTACGACGTGGGCTAC |
| Ms-β-Actin-F | ACAGCAGTTGGTTGGAGCAA |
| Ms-β-Actin-R | ACGCGACCATCCTCCTCTTA |
