# Supplementary material for: Targeted delivery of the PKMYT1 inhibitor RP-6306 mediates PANoptosis in pancreatic cancer via mitotic catastrophe
Source: Cell Death Dis. 2025 Jul 15;16(1):526. doi: 10.1038/s41419-025-07835-2 (PMC12263950; doi:10.1038/s41419-025-07835-2)
Supplement: Supplementary file 4 — Original western blots [file 41419_2025_7835_MOESM4_ESM.pptx]

## Slide 1
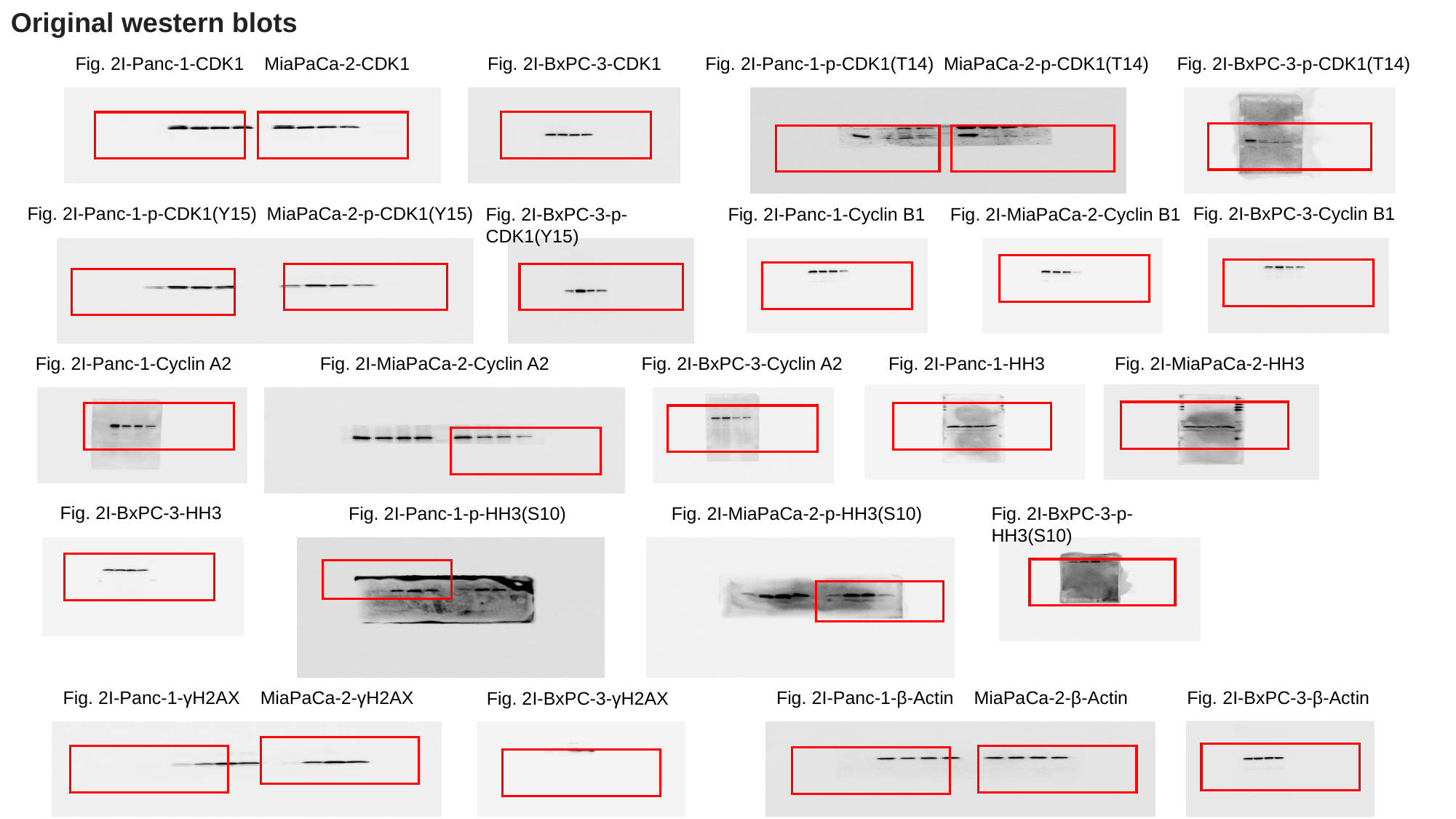

Original western blots
Fig. 2I-BxPC-3-p-CDK1(T14)
Fig. 2I-BxPC-3-CDK1
Fig. 2I-Panc-1-CDK1 MiaPaCa-2-CDK1
Fig. 2I-Panc-1-p-CDK1(T14) MiaPaCa-2-p-CDK1(T14)
Fig. 2I-Panc-1-p-CDK1(Y15) MiaPaCa-2-p-CDK1(Y15)
Fig. 2I-BxPC-3-Cyclin B1
Fig. 2I-MiaPaCa-2-Cyclin B1
Fig. 2I-Panc-1-Cyclin B1
Fig. 2I-BxPC-3-p-CDK1(Y15)
Fig. 2I-MiaPaCa-2-HH3
Fig. 2I-Panc-1-HH3
Fig. 2I-Panc-1-Cyclin A2
Fig. 2I-MiaPaCa-2-Cyclin A2
Fig. 2I-BxPC-3-Cyclin A2
Fig. 2I-BxPC-3-HH3
Fig. 2I-Panc-1-p-HH3(S10)
Fig. 2I-MiaPaCa-2-p-HH3(S10)
Fig. 2I-BxPC-3-p-HH3(S10)
Fig. 2I-Panc-1-γH2AX MiaPaCa-2-γH2AX
Fig. 2I-BxPC-3-β-Actin
Fig. 2I-Panc-1-β-Actin MiaPaCa-2-β-Actin
Fig. 2I-BxPC-3-γH2AX

## Slide 2
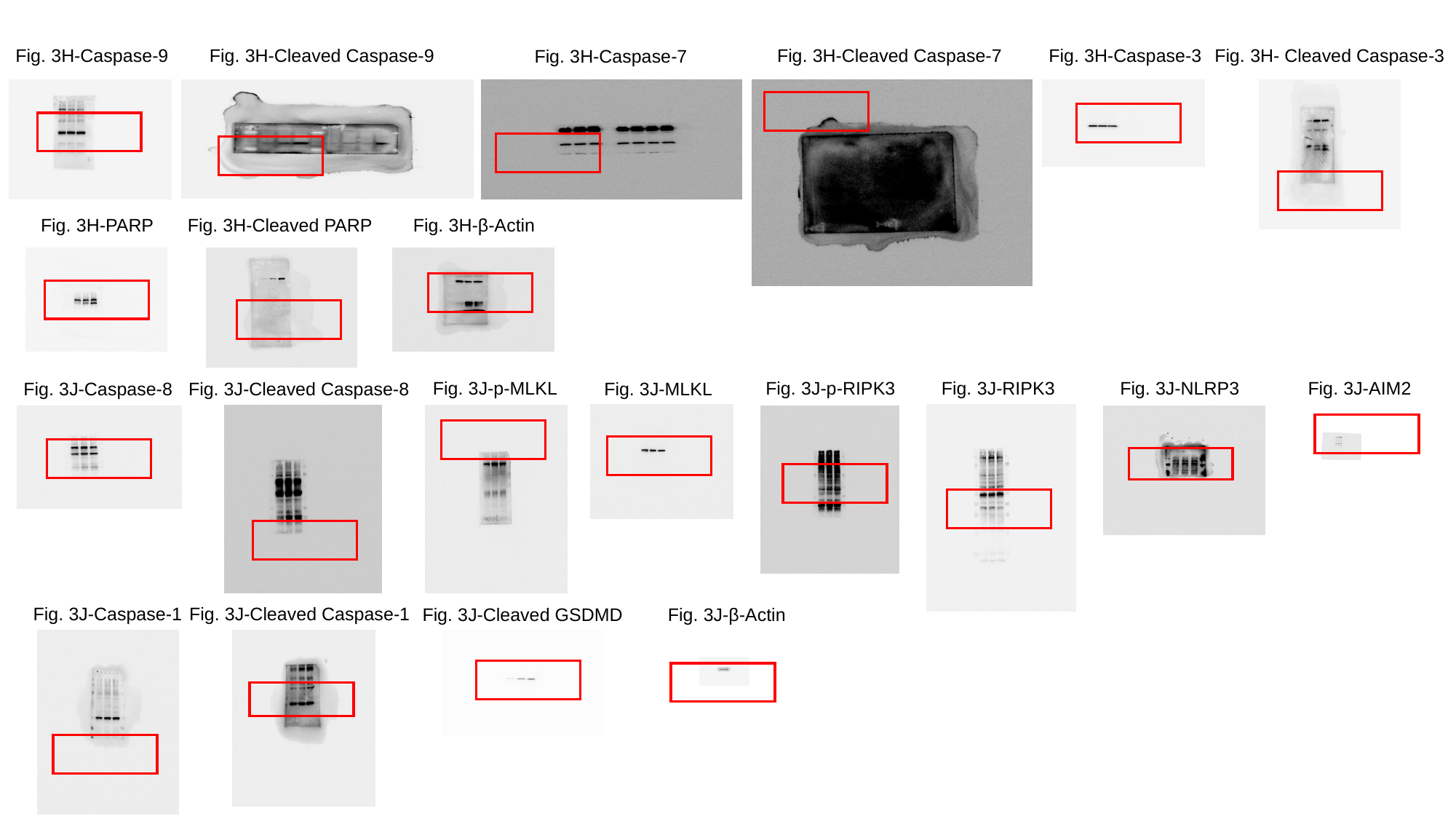

Fig. 3H- Cleaved Caspase-3
Fig. 3H-Cleaved Caspase-9
Fig. 3H-Caspase-3
Fig. 3H-Caspase-9
Fig. 3H-Cleaved Caspase-7
Fig. 3H-Caspase-7
Fig. 3H-Cleaved PARP
Fig. 3H-β-Actin
Fig. 3H-PARP
Fig. 3J-p-RIPK3
Fig. 3J-RIPK3
Fig. 3J-NLRP3
Fig. 3J-AIM2
Fig. 3J-p-MLKL
Fig. 3J-MLKL
Fig. 3J-Caspase-8
Fig. 3J-Cleaved Caspase-8
Fig. 3J-Caspase-1
Fig. 3J-Cleaved Caspase-1
Fig. 3J-Cleaved GSDMD
Fig. 3J-β-Actin

## Slide 3
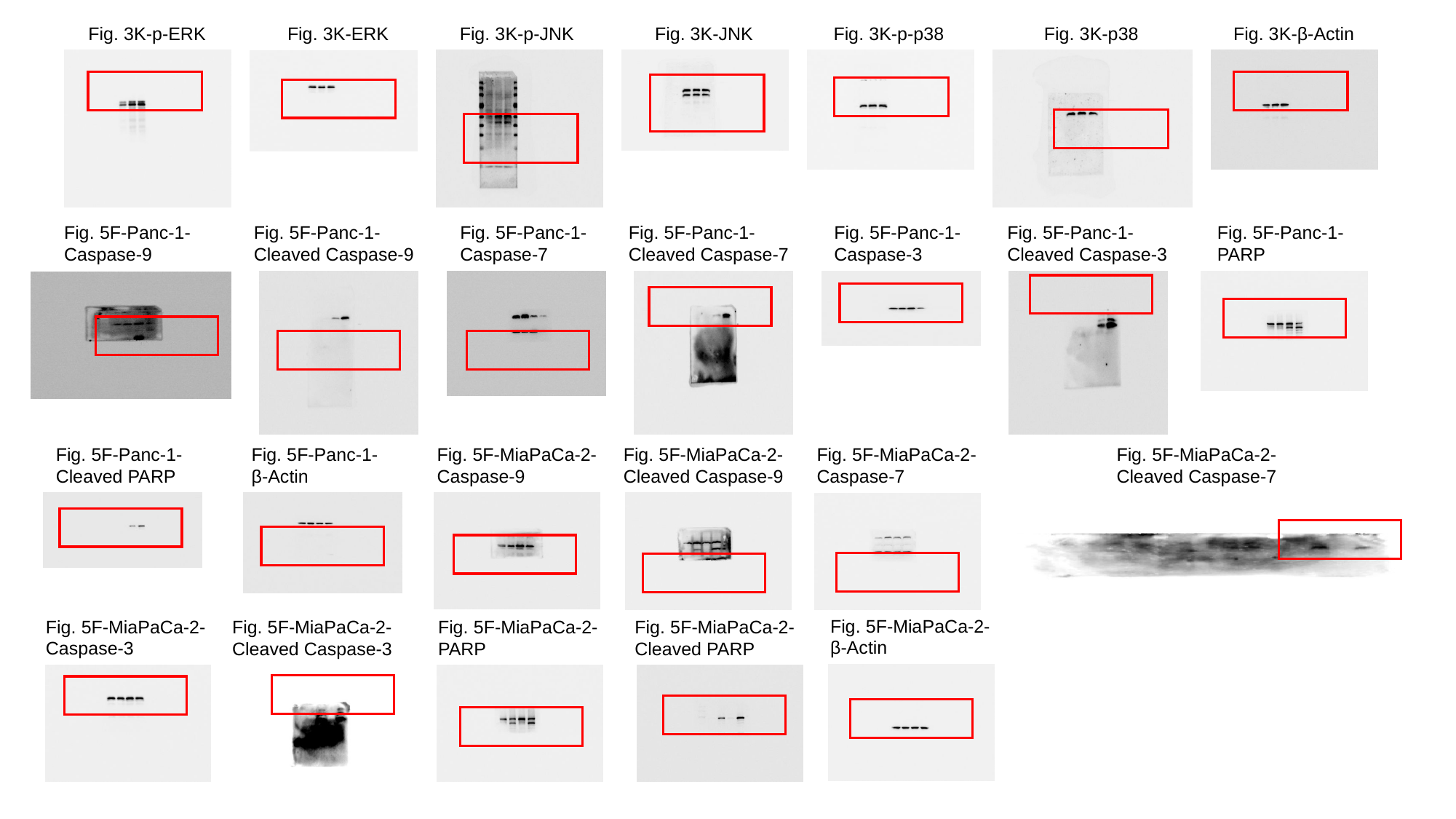

Fig. 3K-p-ERK
Fig. 3K-p-p38
Fig. 3K-p38
Fig. 3K-β-Actin
Fig. 3K-p-JNK
Fig. 3K-JNK
Fig. 3K-ERK
Fig. 5F-Panc-1-Caspase-9
Fig. 5F-Panc-1-Cleaved Caspase-9
Fig. 5F-Panc-1-Caspase-7
Fig. 5F-Panc-1-Cleaved Caspase-7
Fig. 5F-Panc-1-Caspase-3
Fig. 5F-Panc-1-Cleaved Caspase-3
Fig. 5F-Panc-1-PARP
Fig. 5F-Panc-1-Cleaved PARP
Fig. 5F-Panc-1-β-Actin
Fig. 5F-MiaPaCa-2-Caspase-9
Fig. 5F-MiaPaCa-2-Cleaved Caspase-7
Fig. 5F-MiaPaCa-2-Cleaved Caspase-9
Fig. 5F-MiaPaCa-2-Caspase-7
Fig. 5F-MiaPaCa-2-β-Actin
Fig. 5F-MiaPaCa-2-Caspase-3
Fig. 5F-MiaPaCa-2-Cleaved Caspase-3
Fig. 5F-MiaPaCa-2-PARP
Fig. 5F-MiaPaCa-2-Cleaved PARP

## Slide 4
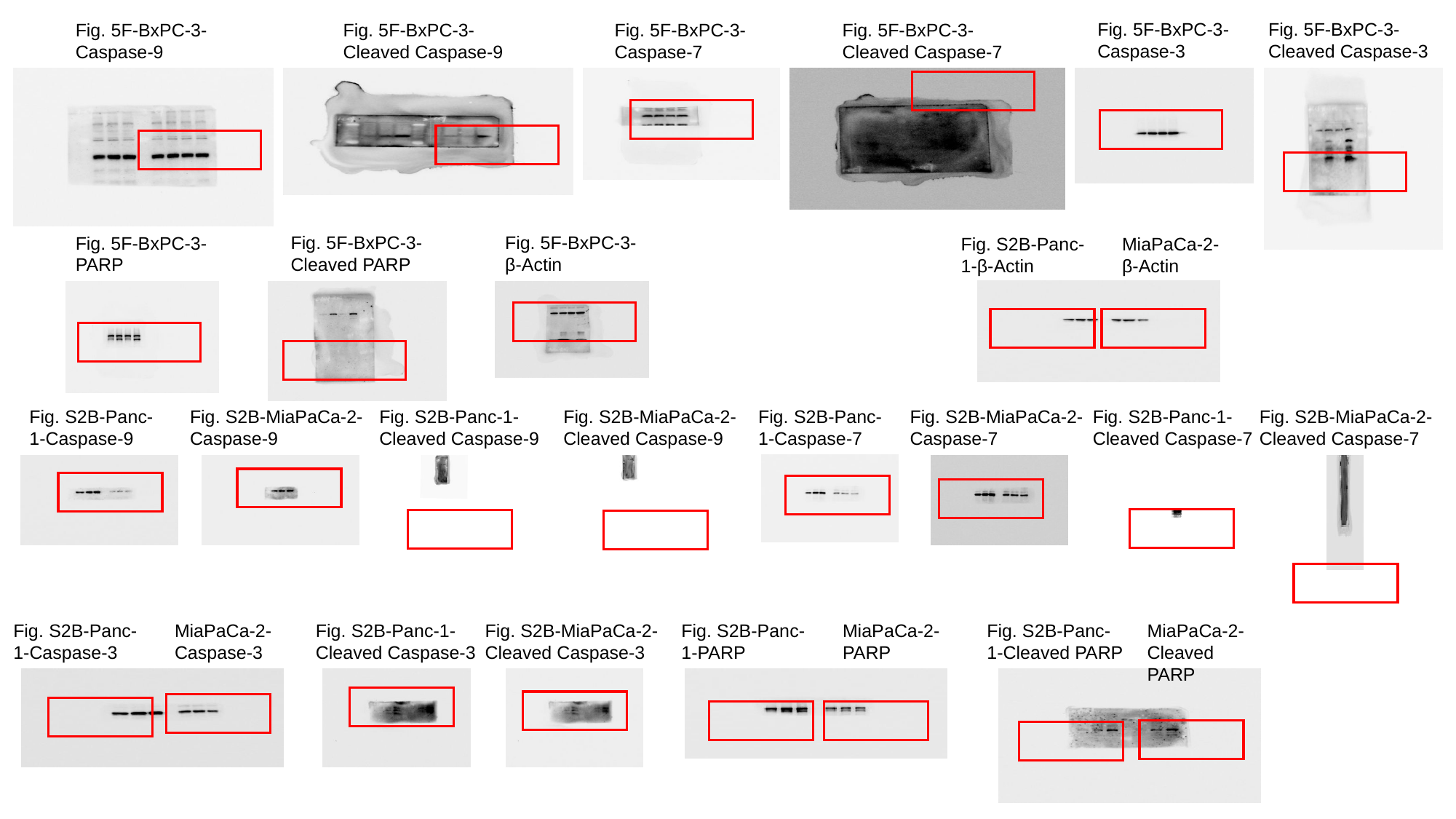

Fig. 5F-BxPC-3-Caspase-3
Fig. 5F-BxPC-3-Cleaved Caspase-3
Fig. 5F-BxPC-3-Cleaved Caspase-7
Fig. 5F-BxPC-3-Caspase-9
Fig. 5F-BxPC-3-Cleaved Caspase-9
Fig. 5F-BxPC-3-Caspase-7
Fig. 5F-BxPC-3-Cleaved PARP
Fig. 5F-BxPC-3-β-Actin
Fig. 5F-BxPC-3-PARP
MiaPaCa-2-β-Actin
Fig. S2B-Panc-1-β-Actin
Fig. S2B-MiaPaCa-2-Cleaved Caspase-7
Fig. S2B-MiaPaCa-2-Caspase-7
Fig. S2B-Panc-1-Cleaved Caspase-7
Fig. S2B-MiaPaCa-2-Caspase-9
Fig. S2B-MiaPaCa-2-Cleaved Caspase-9
Fig. S2B-Panc-1-Caspase-7
Fig. S2B-Panc-1-Caspase-9
Fig. S2B-Panc-1-Cleaved Caspase-9
MiaPaCa-2-PARP
MiaPaCa-2-Cleaved PARP
Fig. S2B-Panc-1-PARP
Fig. S2B-Panc-1-Cleaved PARP
Fig. S2B-Panc-1-Cleaved Caspase-3
Fig. S2B-MiaPaCa-2-Cleaved Caspase-3
Fig. S2B-Panc-1-Caspase-3
MiaPaCa-2-Caspase-3

## Slide 5
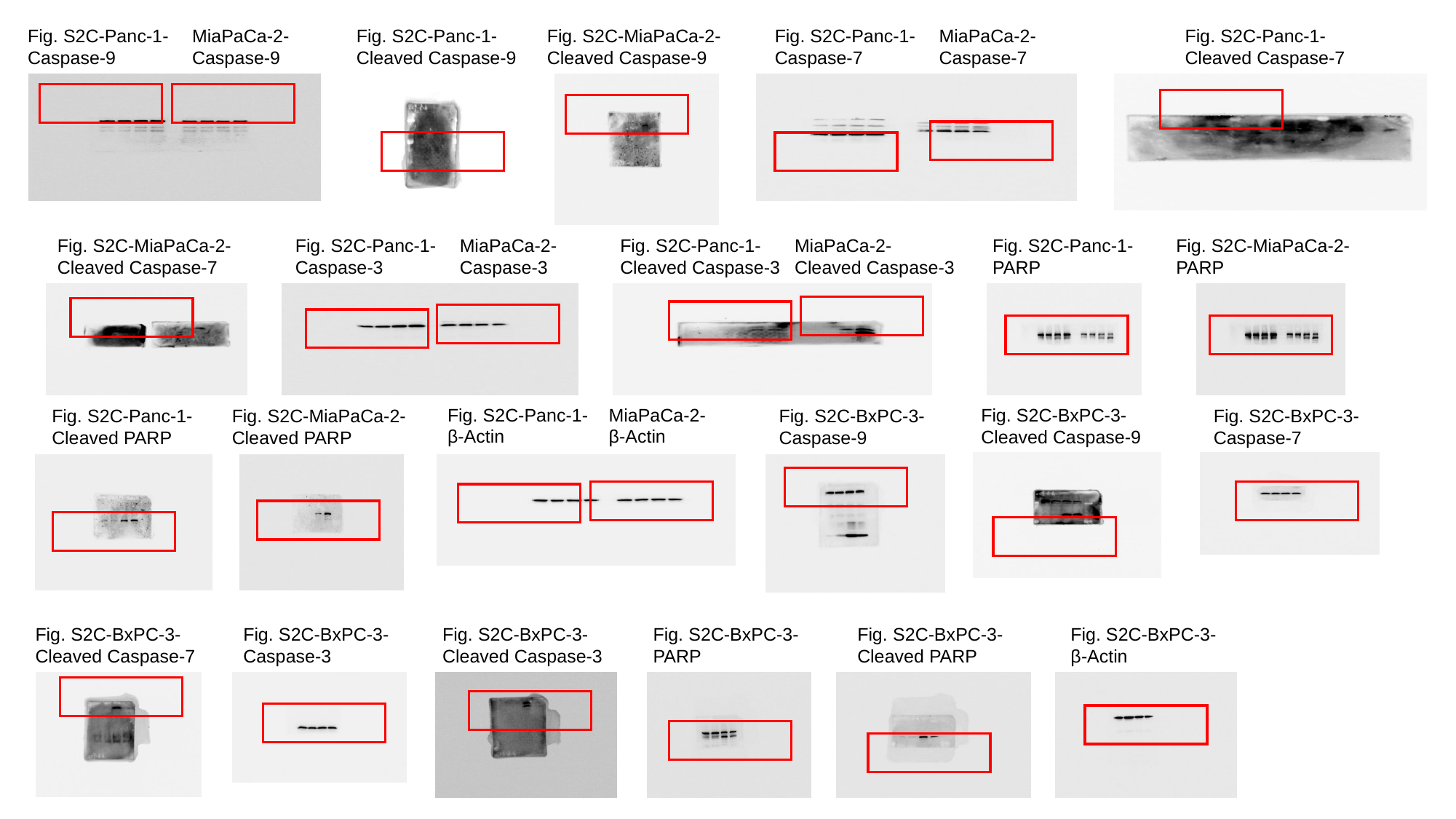

Fig. S2C-Panc-1-Cleaved Caspase-9
Fig. S2C-MiaPaCa-2-Cleaved Caspase-9
Fig. S2C-Panc-1-Caspase-7
MiaPaCa-2-Caspase-7
Fig. S2C-Panc-1-Cleaved Caspase-7
Fig. S2C-Panc-1-Caspase-9
MiaPaCa-2-Caspase-9
Fig. S2C-Panc-1-PARP
Fig. S2C-MiaPaCa-2-PARP
Fig. S2C-Panc-1-Cleaved Caspase-3
MiaPaCa-2-Cleaved Caspase-3
Fig. S2C-MiaPaCa-2-Cleaved Caspase-7
Fig. S2C-Panc-1-Caspase-3
MiaPaCa-2-Caspase-3
MiaPaCa-2-β-Actin
Fig. S2C-Panc-1-β-Actin
Fig. S2C-BxPC-3-Cleaved Caspase-9
Fig. S2C-Panc-1-Cleaved PARP
Fig. S2C-MiaPaCa-2-Cleaved PARP
Fig. S2C-BxPC-3-Caspase-9
Fig. S2C-BxPC-3-Caspase-7
Fig. S2C-BxPC-3-Cleaved Caspase-7
Fig. S2C-BxPC-3-Caspase-3
Fig. S2C-BxPC-3-Cleaved Caspase-3
Fig. S2C-BxPC-3-PARP
Fig. S2C-BxPC-3-Cleaved PARP
Fig. S2C-BxPC-3-β-Actin

## Slide 6
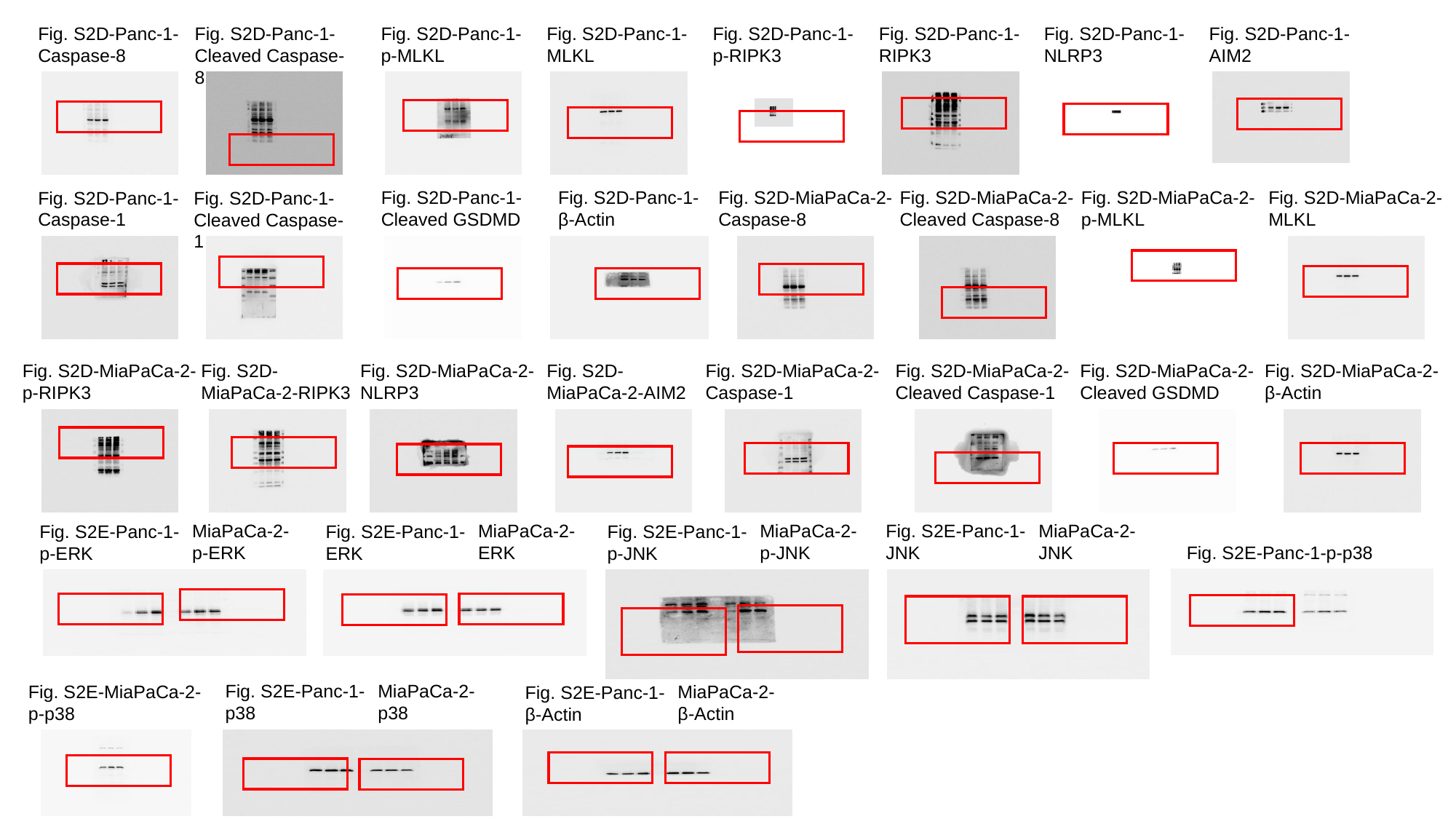

Fig. S2D-Panc-1-p-RIPK3
Fig. S2D-Panc-1-RIPK3
Fig. S2D-Panc-1-NLRP3
Fig. S2D-Panc-1-AIM2
Fig. S2D-Panc-1-Caspase-8
Fig. S2D-Panc-1-Cleaved Caspase-8
Fig. S2D-Panc-1-p-MLKL
Fig. S2D-Panc-1-MLKL
Fig. S2D-MiaPaCa-2-MLKL
Fig. S2D-MiaPaCa-2-p-MLKL
Fig. S2D-MiaPaCa-2-Cleaved Caspase-8
Fig. S2D-MiaPaCa-2-Caspase-8
Fig. S2D-Panc-1-Cleaved GSDMD
Fig. S2D-Panc-1-β-Actin
Fig. S2D-Panc-1-Caspase-1
Fig. S2D-Panc-1-Cleaved Caspase-1
Fig. S2D-MiaPaCa-2-β-Actin
Fig. S2D-MiaPaCa-2-Cleaved Caspase-1
Fig. S2D-MiaPaCa-2-Caspase-1
Fig. S2D-MiaPaCa-2-Cleaved GSDMD
Fig. S2D-MiaPaCa-2-RIPK3
Fig. S2D-MiaPaCa-2-NLRP3
Fig. S2D-MiaPaCa-2-p-RIPK3
Fig. S2D-MiaPaCa-2-AIM2
MiaPaCa-2-JNK
MiaPaCa-2-p-ERK
MiaPaCa-2-ERK
MiaPaCa-2-p-JNK
Fig. S2E-Panc-1-JNK
Fig. S2E-Panc-1-p-ERK
Fig. S2E-Panc-1-ERK
Fig. S2E-Panc-1-p-JNK
Fig. S2E-Panc-1-p-p38
MiaPaCa-2-p38
Fig. S2E-Panc-1-p38
Fig. S2E-MiaPaCa-2-p-p38
MiaPaCa-2-β-Actin
Fig. S2E-Panc-1-β-Actin
